# Comprehensive polyphenolic profiling in promising resistant grapevine hybrids including 17 novel breeds in northern Italy

**DOI:** 10.1002/jsfa.10861

**Published:** 2020-10-26

**Authors:** Verena Gratl, Sonja Sturm, Elena Zini, Thomas Letschka, Marco Stefanini, Silvia Vezzulli, Hermann Stuppner

**Affiliations:** ^1^ Institute of Pharmacy/Pharmacognosy, Center for Molecular Biosciences Innsbruck University of Innsbruck Innsbruck Austria; ^2^ Laimburg Research Centre Vadena Italy; ^3^ Fondazione Edmund Mach, Italy Research and Innovation Centre San Michele all'Adige Italy

**Keywords:** *Vitis*, grapes, resistant varieties, targeted metabolomics, polyphenols, UHPLC–MS/MS

## Abstract

**BACKGROUND:**

A promising way to overcome the susceptibility of *Vitis vinifera* L. to fungal diseases is the integration of genetic resistance by the interspecific crossing between *V. vinifera* varieties and resistant species. However, the products of such hybrids are still not accepted by customers, particularly due to their organoleptic characteristics, not least influenced by their polyphenolic profile.

**RESULTS:**

A total of 58 resistant breeding lines, 41 from international programs and 17 new progeny individuals, were grown in one untreated vineyard to exclude any variances by climatic and pedologic conditions or vineyard practice. A total of 60 polyphenols (including acids, anthocyanins, flavonols, flavan‐3‐ols, and stilbenoids) were determined in grapevine berries by ultrahigh‐performance liquid chromatography–mass spectrometry in two consecutive years. The overall profiles were rather consistent (variation *P* > 0.05) within the two harvests, with the exceptions of epicatechin and caftaric acid. Anthocyanin diglucosides were found in ten of the red breeding lines, malvidin‐3,5‐*O*‐diglucoside being predominant in nine of them. Total polyphenol content of the unknown progeny individuals and international breeding lines was comparable, with the exception of significantly increased amounts of gallic acid and some flavonoids.

**CONCLUSION:**

The comprehensive study reported herein of the polyphenolic profile of hybrids from international breeding programs, but also of new breeds from private initiatives, all cultivated in the same vineyard, will support the selection of promising candidates for further breeding programs to overcome impairment due to undesired sensory characteristics of new highly resistant varieties.

## INTRODUCTION

Grapevine is not only one of the most important horticultural crop species, reflected by ~8 × 10^6^ ha of vineyards worldwide, 43% thereof in Europe, but is also one of the oldest. The history of cultivation of the predominant domesticated species *Vitis vinifera* L. can be traced back more than 6000 years.^1^, ^2^, ^3^ Centuries of cultivation efforts, mainly by vegetative propagation and crossing activities, have resulted in several hundred high‐quality cultivars that are in use nowadays. However, for more than 150 years, when grape phylloxera (caused by the insect *Daktulosphaira vitifoliae*) and downy mildew (caused by the oomycete *Plasmopara viticola*) were introduced from North America into Europe, a sophisticated pest and pathogen control has been mandatory for successful viticulture. Whereas breeders managed to gain control over grape phylloxera, the susceptibility of *V. vinifera* against *P. viticola*, as well as *Erysiphe necator* (syn. *Uncinula necator*), the ascomycete causal agent of powdery mildew, remains problematic in viticulture. A widely accepted and eco‐friendly strategy for reduction of necessary treatments with fungicides is the integration of genetic resistance by interspecific breeding between *V. vinifera* and North American or Asian resistant species.^3^, ^4^, ^5^ Modern breeding programs aim at the development of new lines with resistance against pests and pathogens, but also with winter hardiness and resistance against early or late frosts, sufficient drought tolerance, and excellent quality traits. However, with the exception of a few approved cultivars, acceptance of resistant hybrids is often hampered by undesirable sensory characteristics, such as high acidity, low astringency, or so‐called off‐flavors.

A big disadvantage of conventional breeding is the tremendous amount of time required to produce results. Detection of genetic markers and genetic engineering can be used to assist in speeding up the selection of promising highly resistant breeds, and profiling approaches can provide insight into the composition of secondary metabolites in grapes, or products thereof. One class of constituents with an enormous impact on the overall properties of grapes, and particularly of wine, the most important product thereof, are polyphenolic compounds. Polyphenols are a structurally heterogenous class of aromatic compounds, with usually two or more phenolic hydroxyl functions. Exceeding a certain chromophore size, their color ranges from yellow to red or dark blue. The phenolic functions make these compounds participators in redox reactions, hence facilitating the chemical interaction with other organic compounds. In addition, owing to their aromaticity, polyphenols are very effective radical scavengers. Polyphenols are responsible for the coloration of wine, and to some extent participate in the wine odor, either directly or indirectly in the aging process of wine. Besides their influence on the appearance of wine, their contribution to the taste sensations of the consumer is of importance. Polyphenol congeners contribute to taste sensations such as sweet, acidic, and bitter, and they attribute to impressions such as velvety, smooth, or astringent in our mouth.^3^, ^6^, ^7^ In modern society, however, the health benefits attributed to polyphenols seem to be of higher importance than their contribution to the organoleptic impression of the product ‘wine’. Besides their distinct antioxidant activity, congeners of this substance class have been shown to be cardio‑ and hepatoprotective and to possess anti‐thrombotic, anti‐inflammatory, anti‐allergic, or anti‐carcinogenic activities.^8^, ^9^, ^10^, ^11^, ^12^ They are also pivotal for plants regarding defenses against biotic and abiotic stresses, acting as protectors against UV radiation, herbivores, parasites, or diseases or by restricting the growth of neighboring plants.^11^, ^13^, ^14^ The accumulation of those compounds in the berries is highly influenced by several factors, such as environmental conditions, climate, light, temperatures, humidity, or viticulture practices.^7^, ^14^, ^15^, ^16^

The overall polyphenol metabolite profiles, however, vary considerably among different cultivars and are under strict genetic control. Thus, they have been shown to represent useful markers for the authentification of grapes and wines.^17^, ^18^, ^19^

The polyphenolic composition of *V. vinifera* grapes was intensively studied during the last few decades.^16^, ^17^, ^19^, ^20^, ^21^, ^22^ In recent years, several studies were published investigating the chemical composition of species/genotypes other than *V. vinifera* ^3^, ^7^, ^23^, ^24^ and grapevine hybrids,^24^, ^25^, ^26^, ^27^, ^28^, ^29^, ^30^, ^31^, ^32^ though in all cases limited to a few accessions or one subclass of polyphenols.

About a decade ago, an ambitious breeding and cultivation project aiming at the production of new mildew‐resistant varieties lacking negative quality traits was initiated. A total of 58 genotypes, 41 highly resistant breeds and 17 new and uncharacterized progeny individuals (PIs), were grown in an untreated vineyard located in Marlengo, South Tyrol. The aim of the VITISANA project was to comprehensively characterize these breeds independent of influences by macroclimatic and pedologic conditions or vineyard practice. The analytical strategy chosen was to utilize an already well established and validated liquid chromatography (LC)–tandem mass spectrometry (MS/MS) analytical procedure capable of quantifying dozens of polyphenolic congeners in combination with a strict harvesting plan aiming at the exclusion of biological confounding factors. Harvests of two consecutive years were investigated to address the need for biological replicates, since only single plant (vine) analysis was mandatory to ensure genetic uniformity of the novel accessions. For comparability between the different breeds, ripeness of the grapes was monitored carefully, and only ripe clusters were processed for further analysis. To represent the biological range of berry growth on a vine, three individual clusters were harvested and berries from all clusters were processed.

**Table 1:** Concentrations (mg kg^−1^ fresh weight) of anthocyanin diglucosides detected in grape berries

Accession name	Mv‐3,5‐*O*‐diglc	Dp‐3,5‐*O*‐diglc	Cn‐3,5‐*O*‐diglc	Pt‐3,5‐*O*‐diglc	Pn‐3,5‐*O*‐diglc	Sum
*2017*						
COARNA N. × PIERELLE × SV 20366	8.38	n.d.	2.35	3.00	20.75	34.48
BRUSKAM	385.29	52.65	6.92	116.68	71.38	632.92
CABERNET CORTIS	195.52	32.10	6.48	70.18	31.25	335.52
LEON MILLOT	438.34	109.26	23.52	183.02	50.05	804.19
LU 1	339.53	205.31	72.53	393.95	147.36	1158.68
LU 2	1062.75	351.56	47.24	721.98	200.11	2383.64
IV045	20.33	n.d.	0.31	3.96	3.36	27.97
SEMONELL	173.64	40.76	12.21	87.22	93.39	407.23
IV039	355.40	51.03	12.24	123.82	113.60	656.09
IV035	176.42	5.66	1.00	11.82	66.47	261.37
*2018*						
COARNA N. × PIERELLE × SV 20366	2.83	n.d.	1.00	1.11	7.85	12.79
BRUSKAM	618.50	35.02	3.65	107.85	50.60	815.62
CABERNET CORTIS	290.50	28.28	4.79	70.23	26.48	420.26
LEON MILLOT	820.50	161.68	50.79	409.00	75.10	1517.07
LU 1	675.50	231.72	35.05	413.00	109.45	1464.72
LU 2	1236.00	219.81	41.62	570.50	139.51	2207.43
IV045	34.99	n.d.	n.d.	4.13	3.52	42.63
SEMONELL	253.00	36.43	8.39	87.33	45.10	430.24
IV039	615.50	29.15	8.17	100.39	118.57	871.77
IV035	259.50	5.68	n.d.	17.87	105.08	388.12

Abbreviations: Mv, malvidin; Dp, delphinidin; Cn, cyanidin; Pt, petunidin; Pn, peonidin; diglc, diglucoside.

## MATERIAL AND METHODS

### Plant material

Fifty‐eight grapevine accessions were grown in an untreated vineyard located in a private winery in Marlengo (BZ, Italy) (46.670938°N, 11.131313°E, 401 m a.s.l.). Forty‐one thereof were breeding lines (BLs) originating from various European breeding programs, and 17 lines were new PIs from a private breeder (InnoVitis, Marlengo, South Tyrol (BZ), Italy). Detailed information for all accessions, including variety number VIVC, age of vine, medium yield, species involved, and origin of mildew resistance is provided in Supporting Information Table S1. The accessions were managed since 2010 using a Guyot training system with a planting density of 2 m × 0.8 m in terraced fields. All accessions were grafted on SO4 rootstocks (*Vitis berlandieri* × *Vitis riparia*), grown in loamy sand (see Supporting Information Table S2). Weather conditions, such as humidity, precipitation, hours of sunshine, or wind conditions, were recorded by a weather station in proximity to the vineyard and can be accessed on the website (http://meteobrowser.eurac.edu/app_direct/meteobrowser) for the Meran/Merano station (distance to the vineyard about 2 km). In 2017 and 2018, at least three clusters per single accession (about 1000 g) were harvested from one plant at maturity level (18 °Bx). The plant material was frozen in liquid nitrogen, transported on ice, and stored at −80 °C until analysis.

Rosé‐colored accessions were considered as white‐skinned grapes in statistical evaluation and figures; all red accessions were of skin color ‘noir’ (see Supporting Information Table S1).

### Extraction of metabolites

Extraction was performed as described by Vrhovsek *et al*.^33^ Briefly, 200 g of frozen grape berries (representative collection from all frozen clusters) were ground using an IKA analytical mill (Staufen, GER) under liquid nitrogen and 2 g of the frozen powder was extracted using a mixture of water–methanol–chloroform. The aqueous layer was diluted to 10.00 mL and filtered (0.2 μm polytetrafluoroethylene filter) into high‐performance LC vials. As quality control samples, extracts from eight cultivars were prepared in triplicate.

### Chromatographic conditions

Ultra‐performance LC (UPLC) analysis was carried out on an Acquity UPLC system (Waters, Milford, MA, USA) coupled to a Xevo TQMS System (Waters) with an electrospray ionization source. Phenolic compounds (except anthocyanins) were analyzed according to Vrhovsek *et al*.^33^ using an Acquity HSS T3 column (100 × 2.1 mm, 1.8 μm; Waters), and a mobile phase consisting of water (A) and acetonitrile (B), both with 0.1% formic acid, at a flow rate of 0.4 mL min^−1^. The temperature was kept at 40 °C. The gradient profile was 0 min 5% B, from 0 to 3 min to 20% B, from 3 to 4.3 min isocratic 20% B, from 4.3 to 9 min to 45% B, from 9 to 11 min to 100% B, from 11 to 13 min at 100% B, and from 13.01 to 15 min back to 5% B. Injection volume was 2 μL. Mass spectrometric conditions were as follows. Capillary voltage was +3.5 kV and −2.5 kV in positive and negative mode respectively. The source was kept at 150 °C, and the desolvation temperature was 500 °C. Cone gas flow was 50 L h^−1^, and desolvation gas flow was 800 L h^−1^. The dwell time was ≥25 ms for each multiple reaction monitoring event.

Anthocyanins were analyzed according to Arapitsas *et al*.^34^ using an Acquity UPLC BEH C18 column (2.1 mm × 150 mm, 1.7 μm; Waters) and an Acquity UPLC BEH C18 precolumn (2.1 mm × 5 mm, 1.7 μm; Waters) at 40 °C. The mobile phase was water (A) and methanol (B), each with 5% v/v formic acid. The gradient was 0 min 5% B, from 0 to 4 min linear to 40% B, from 4 to 9 min linear to 55%, from 9 to 11 min linear to 95% B, and from 11 to 14 min back to the initial conditions of 95% B. The injection volume was 2 μL. Mass spectrometric conditions were as follows. Electrospray ionization in the positive ionization mode with a capillary voltage of 0.5 kV, block temperatures of 150 °C, and desolvation temperature of 500 °C. Cone gas flow was 20 L h^−1^, and desolvation gas flow was 1000 L h^−1^.

Peak identification, optimization of multiple reaction monitoring events, and quantitation were performed with standards as described by Vrhovsek *et al*.,^33^ with concentrations calculated in milligrams per kilogram of grape berry fresh weight (FW). The 3‐*O*‐galactosides of quercetin and syringetin were calculated using the calibration of their respective 3‐*O*‐glucosides, and procyanidin B3 was calculated with the calibration of procyanidin B1. All data reported did exceed the limit of detection figures of the assay.^33^ Analytes not exceeding this threshold were reported as ‘not detected’.

### Data analysis

Data were processed using Waters MassLynx (version 4.1) and TargetLynx software (Waters). Data analysis and production of graphs, including one‐way analysis of variance, box‐plots, and heatmaps, were performed using R software (version 3.3.3 R Development Core Team, Vienna, Austria, 2018), Excel 2016 (Microsoft Office Professional Plus 2016), and SPSS Statistics software, version 24.0 (IBM Corporation, Armonk, NY, USA). For principal component analysis (PCA), SIMCA 13.0 software was used (Umetrics, Umeå, Sweden). Unit‐variance scaling and mean centering were applied for heatmaps and PCA, and compounds with more than 50% missing values were excluded in the heat maps. Dendrograms were constructed by hierarchical clustering analysis using ‘Canberra’ as the distance function and Ward.D2 as the hierarchical clustering algorithm.

## RESULTS AND DISCUSSION

### Phenolic profiles

Following the strategy to lower agro‐chemical input by promoting mildew‐resistant varieties, almost 100 novel plants derived from crossing resistant genotypes and quality varieties were cultivated in an untreated vineyard in South Tyrol. Fifty‐eight accessions (41 highly resistant breeds and 17 new progeny lines – for details see Supporting Information Table S1), bearing enough grapes for harvests in two consecutive years, were selected for a comprehensive genotypic and phenotypic characterization. Within this study, quantitative profiling of phenolic compounds in grapes (in total 60 derivatives, such as hydroxycinnamic and hydroxybenzoic acids, stilbenoids, flavan‐3‐ols, flavonols, anthocyanins, and others) was conducted by a targeted and validated LC–MS/MS assay.

Since the general figures such as limit of quantitation and analytical repeatability of merit of the applied assay were already published as part of the method validation, repeated sampling was not performed in that investigation.^33^ Consequently, the sampling strategy and the measurement campaign presented here was accompanied by experiments allowing a deeper insight into the total variability of the data generated. From the pooled grapes (see Material and Methods section for details), three subsamples were picked, prepared, and measured. Mean values, standard deviations, and relative standard deviations (RSDs) were calculated. A total of 220 data points was generated: 60 for anthocyanins (A‐type) and 160 for other polyphenols (P‐type) (Supporting Information Table S5). For A‐type analytes (median concentration 27.2 mg kg^−1^) a median RSD of 9.2% was found, with 80% of all RSD values below 14.5%. For P‐type analytes (median concentration 15.7 mg kg^−1^) a median RSD of 17.2% was found, with 80% of all RSD values below 28%. These data are in agreement with the data presented in the validation study,^33^ where, in a single analysis of a grape sample, RSD values of less than 20% were realized for 80% of all analytes. It is reasonable to argue that repeated sampling and repeated analysis expand this figure of merit due to the law of error propagation.

**Figure 1:**
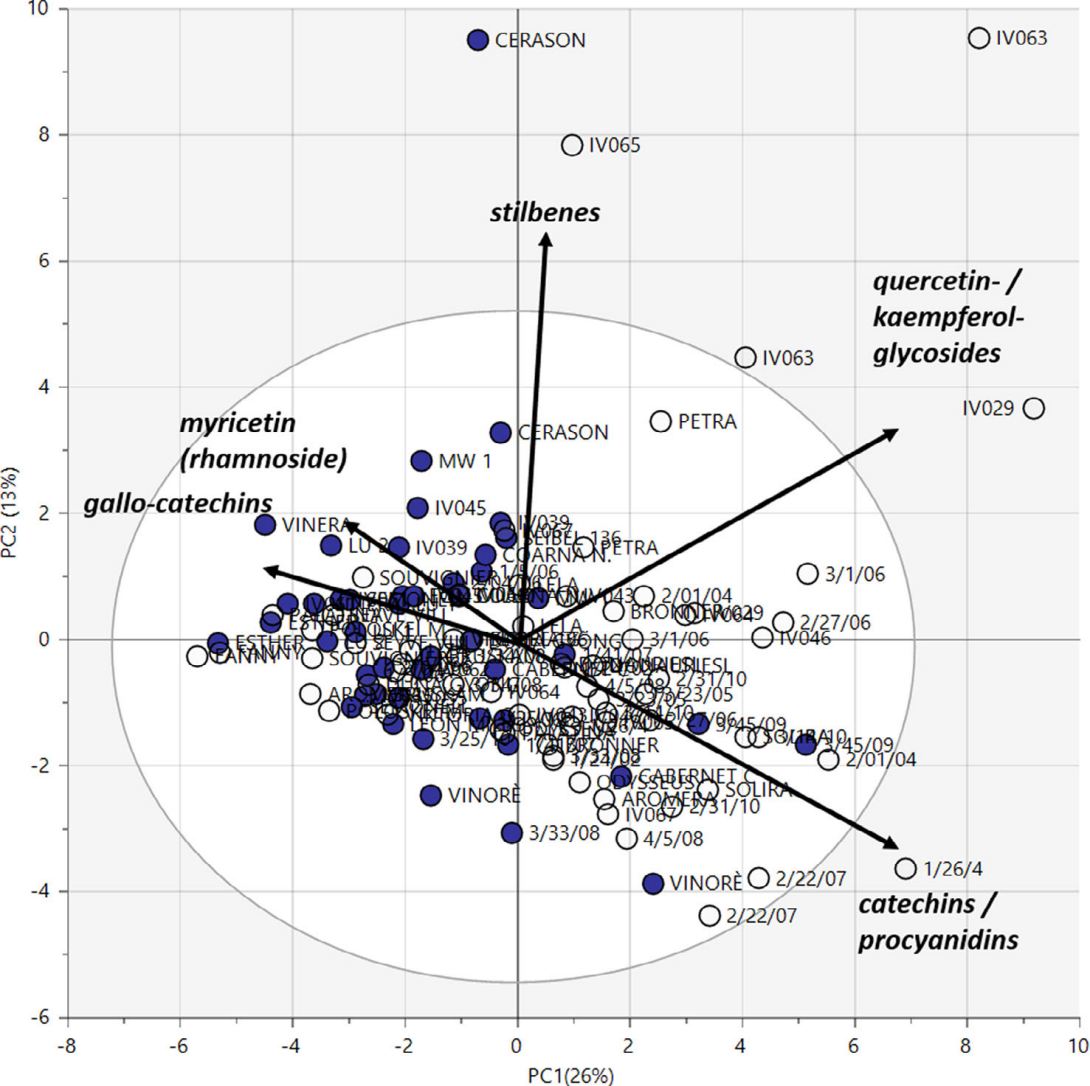
PCA of the polyphenolic profile (anthocyanins excluded) of all 58 accessions (filled circles are red grapes, empty circles are white accessions).

Based on the derived quantitative data, multivariate statistical analysis was performed. A PCA provided a first overview. No clear grouping of the 2017 and 2018 harvests or white and red grapes was observed when anthocyanins were excluded (Fig. 1). However, catechins, procyanidins, and quercetin and kaempferol glycosides in white grapes and gallocatechins and the presence of myricetin and its 3‐*O*‐rhamnoside in red grapes contributed to the moderate differentiation between white and red grapes (see Supporting Information Figs S1 and S2).

The red accession ‘Cerason’ was an outlier in 2018 due to a tenfold increase in stilbenoids (particularly *trans*‐resveratrol and piceatannol), whereas the white grapes of the cultivar ‘IV065’ did show >90% elevated levels of the stilbenoids *trans*‑ and *cis*‐piceide and of arbutin in 2017. Cultivar ‘IV063’ was close to the 95% confidence interval in 2017 and was a clear outlier in 2018 due to approximately twofold concentrations of the same stilbenoids and a moderate increase in kaempferol glycosides. The cultivar ‘IV029’ was observed as an outlier in 2018 due to increased amounts of flavan‐3‐ols.

The quantitative results for the 60 phenolic compounds are summarized in Supporting Information Table S3 (chemical groups) and given in more detail in Supporting Information Table S4 (single derivatives). The total amount of all compounds addressed ranged from 182.2 mg kg^−1^ FW to 2065.0 mg kg^−1^ FW in white grapes and, due to the additional content of anthocyanins, from 745.7 mg kg^−1^ FW to 7076.1 mg kg^−1^ FW in red accessions.

As already shown qualitatively in the PCA (Fig. 1), no statistically significant (*P* > 0.05) differences in the phenolic profiles between the harvests in 2017 and 2018 were observed with a very few exceptions (see Supporting Information Tables S6 and S8). The median amount of epicatechin was lower in 2018, it decreased from 146.4 mg kg^−1^ FW to 74.0 mg kg^−1^ FW in red grapes (*P* = 0.01) and from 136.8 mg kg^−1^ FW to 72.1 mg kg^−1^ FW in white grapes (*P* = 0.002). Levels of caftaric acid were comparable in 2017 in red and white grapes (28.3 mg kg^−1^ FW and 28.7 mg kg^−1^ FW respectively) but were markedly (*P* = 0.01) elevated to 51.0 mg kg^−1^ FW in 2018 in white grapes. Although a great variance was observed between years for most accessions when looking at single compounds, no significant differences were detected considering the overall phenolic profile (*P* > 0.1).

#### *Phenolic acids*

**Figure 2:**
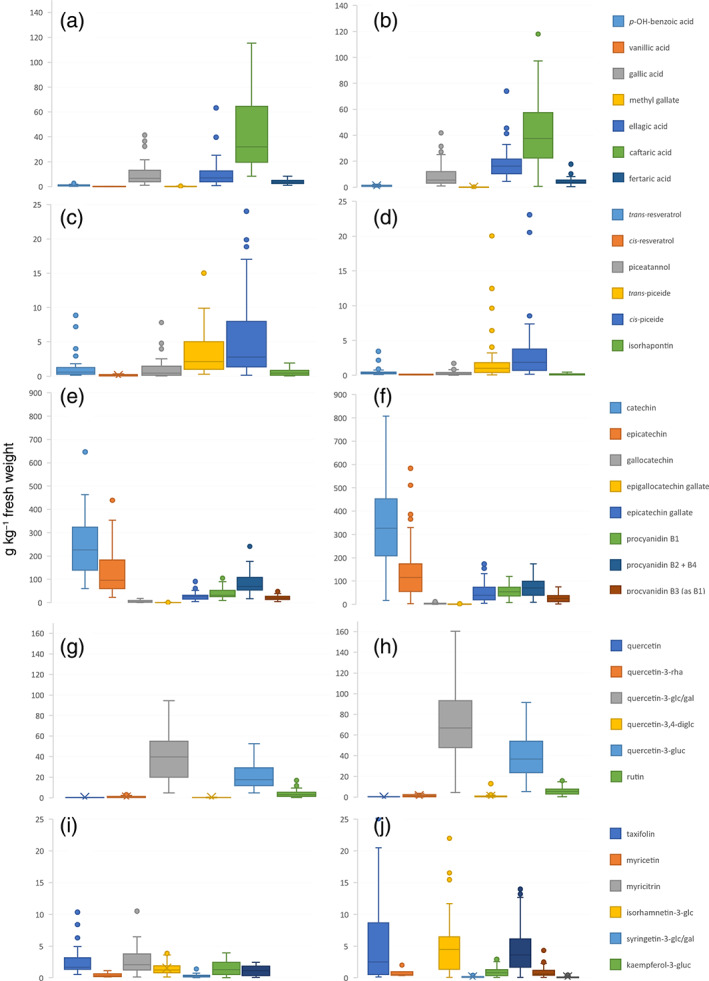
Box plots illustrating the content and distribution of polyphenols, hydroxybenzoic acids, and hydroxycinnamic acids in (A) red and (B) white, stilbenes in (C) red and (D) white, flavan‐3‐ols in (E) red and (F) white, flavonols in (G, I) red and (H, J) white accessions. Description of boxes: median, central line; mean, cross; interquartile range box, 25th to 75th percentile; whiskers, <1.5 times interquartile range; outliers marked as filled circles.

Seven phenolic acids were quantified, the contents and distribution are shown in Fig. 2(A)for red berries and Fig. 2(B) for white berries. The median amount of hydroxybenzoic acids was 16.6 mg kg^−1^ FW in red berries and 31.4 mg kg^−1^ FW in white grapes. When Liang *et al*.^22^ studied the phenol content in 344 European *V. vinifera* grapes, vanillic acid accounted for about 70% of all acids, with amounts up to 43 mg kg^−1^ FW in wine grapes and up to 76 mg kg^−1^ FW in table grapes. In our study, vanillic acid could only be detected in a few red accessions (<15 mg kg^−1^ FW). The dominating benzoic acids in all samples were gallic acid and its condensed dimer ellagic acid, the former with amounts of 1.5–36.6 mg kg^−1^ FW in red grapes and 0.9–41.9 mg kg^−1^ FW in white grapes and the latter with amounts of 0.8–63.3 mg kg^−1^ FW in red grapes and 5.4–74.1 mg kg^−1^ FW in white grapes. In recent years, ellagic acid in particular has been a focus of interest owing to its various pharmacological properties. In a recently published review, ellagic acid was even discussed as a potential lead for novel therapeutics.^35^ From a structural point of view, it is the phenolic acid core unit of ellagitannins and hydrolysable tannins – often conjugated to sugar moieties. It is a very abundant secondary metabolite structural motif and is isolatable from many plant sources, including any tannin‐rich berries. Regarding grapes, its presence is well established; notably, Narduzzi *et al*.^3^ observed that it accumulates to a much higher degree in the skin of grapes of wild American species than in *V. vinifera*.

Hydroxycinnamic acids and their esters are the dominant phenolics in white wines and, besides flavonoids, also in red ones.^18^ Fertaric acid is considered as a precursor for 4‐vinyl‐guaiacol in wine, a negative quality trait when present in high amounts.^30^ Within this study, low and comparable amounts of fertaric acid were found in both years in white and in red grapes (median 3.9 mg kg^−1^ FW in white grapes and 3.7 mg kg^−1^ FW in red grapes). Notable higher concentrations were detected in three white breeds only (‘IV064’: 9.0 mg kg^−1^ FW; ‘IV029’: 14.5 mg kg^−1^ FW; and ‘Lela’: 10.8 mg kg^−1^ FW). Levels of caftaric acid were, as already discussed, comparable in 2017 in red and white grapes but significantly elevated in white grapes in 2018.

#### *Stilbenoids*

Stilbenoids are well‐known constituents in grapes, and also in several other edible berries, due to their role in plant defense, as allelochemicals, and for their interesting health‐related characteristics.^36^, ^37^, ^38^ *trans*‐Resveratrol and its *cis*‐isomer, its glucosides *trans*‑ and *cis*‐piceide, the hydroxylated derivative piceatannol, and isorhapontin were quantified in this study.

Red accessions showed a higher median content and wider distribution of total stilbenoids (median amount 6.6 mg kg^−1^ FW) than white accessions did (3.1 mg kg^−1^ FW), and new BLs had a higher mean level of stilbenoids (10.5 mg kg^−1^ FW) than progeny lines (5.4 mg kg^−1^ FW) (see Supporting Information Tables S7 and S9). The most abundant derivatives in all accessions were the two piceid isomers (see Fig. 2(C), (D)). As already seen qualitatively in the PCA (Fig. 1), ‘IV063’ contained increased levels in 2018, with 36.6 mg kg^−1^ FW and 57.7 mg kg^−1^ FW of *trans*‑ and *cis*‐piceide respectively (2017: 12.5 mg kg^−1^ FW and 23.1 mg kg^−1^ FW); in ‘IV065’, on the other hand, the *trans*‑ and *cis*‐piceide contents decreased from 20.0 mg kg^−1^ FW and 52.5 mg kg^−1^ FW respectively in 2017 to 0.9 mg kg^−1^ FW and 3.7 mg kg^−1^ FW respectively in 2018. The red grapes of ‘Cerason’ contained the highest amount of total stilbenoids in 2018 (47.9 mg kg^−1^ FW) and an extraordinary high level of *trans*‐resveratrol (8.9 mg kg^−1^ FW) and piceatannol (7.8 mg kg^−1^ FW) compared with all other breeds. Although those increased stilbenoid levels correlate with observed levels of mildew infection in leaves and clusters of ‘Cerason’ (International Organization of Vine and Wine scores ≤3 for downy mildew and ≤5 for powdery mildew) and could be discussed as host response to the fungal attack, utmost care must be taken not to overlook possible other triggers contributing to stilbenoid formation. In contrast to the report of Pedneault and Provost,^4^ no correlation between the stilbenoid spectrum and the originating country (Austria, Czech Republic, France, Germany, Hungary, Italy, Russia, Serbia, Switzerland, Ukraine, or Uzbekistan) of the novel breeds was observed.

#### *Flavan‐3‐ols*

Flavan‐3‐ols, beside anthocyanins in red grapes, the most abundant class of polyphenols and mainly present in skin and seeds, do play an important role in wine production, accounting for sensory attributes such as bitterness and astringency. Both monomeric flavan‐3‐ols (e.g. (+)‐catechin, (−)‐epicatechin, (+)‐gallocatechin) and dimeric forms (procyanidin B1, procyanidin B2 + B4, and procyanidin B3) were quantified in the present study.

White accessions were insignificantly (*P* = 0.12; see Supporting Information Table S7) richer in flavanols than red accessions in both years, with median values of 710.4 mg kg^−1^ FW in 2017 and 711.6 mg kg^−1^ FW in 2018, compared with red accessions with median values of 519.4 mg kg^−1^ FW in 2017 and 483.3 mg kg^−1^ FW in 2018 (see Fig. 2(E), (F)).

As reported in other studies,^28^, ^30^, ^39^ (+)‐catechin was by far the most abundant flavanol in all accessions. In contrast to the total sum of flavanols, significant differences (*P* < 0.05) were found between the median content in white grapes (327.9 mg kg^−1^ FW) and red grapes (223.5 mg kg^−1^ FW), with insignificant variation between years. The situation was different for (−)‐epicatechin, however, where there was a noteworthy drop in the median amount to about half, as discussed earlier.

Condensed catechin and/or epicatechin derivatives represent a further prominent group of flavanols which are considered as the main oxidants in grapes.^3^ In our study, the dimer procyanidins (B1–B4) accounted for 12.7–50.1% of the total flavanols; the ratio of monomeric to dimeric derivatives was approximately 3:1 (0.9–6.2:1).

#### *Flavonols*

In contrast to the group of flavanols, white grapes contained significantly (*P* < 0.001; see Supporting Information Table S7) higher amounts of flavonols – more than 90% thereof quercetin glycosides – than red ones in both years (median amounts of 127.8 mg kg^−1^ FW *versus* 70.5 mg kg^−1^ FW). The flavonol‐richest accession was ‘IV029’, with a total of 331.0 mg kg^−1^ FW in 2018 (see Fig. 2(G)–(J)).

This finding is plausible, since all flavonoid analyte classes studied – such as flavanols, flavonols, and also anthocyanins present in red grapes only – originate from the same key molecules in the flavonoid biosynthesis pathway.^13^

Red‐colored grapes, on the other hand, were characterized by the presence of myricetin, myricetrin, and syringetin glycosides, which were found in all red accessions (median amount 4.2 mg kg^−1^ FW) but only in traces in white grapes in a very few cases (see Fig. 2(I), (J)). In contrast to other studies,^30^, ^39^ taxifolin was detected in almost all accessions, and isorhamnetin glycoside was more prominent in white grapes (4.5 mg kg^−1^ FW *versus* 2.1 mg kg^−1^ FW).

#### *Others*

Four further phenolic compounds, not so well known as grape constituents but already reported to be present in *V. vinifera* and American genotypes,^7^ were addressed in our study; namely, the 7‐*O*‐glucosides of luteolin and naringenin, the dihydrochalcone phlorizin, and the hydroquinone derivative arbutin. No significant differences were observed between the two years or the color of grapes (see Supporting Information Tables S3, S6–S8).

#### *Anthocyanins*

Anthocyanins are not only of interest as color‐giving pigments in many fruits and flowers, but also because of their proven benefits on our health.^40^ As reported by Balík *et al*.,^29^ the relative distribution of single compounds within this class is characteristic for each variety and remains more or less constant; changes can be observed in the overall content.

As to be expected, anthocyanins were found in all 23 red accessions in varying amounts (in total 269.7–6148.1 mg kg^−1^ FW), to some extent also in grapes classified as rosé colored (29.3–84.4 mg kg^−1^ FW), and, surprisingly, in two white accessions, namely ‘IV067’ (24.8 mg kg^−1^ FW) and ‘Souvignier gris’ (45.5 mg kg^−1^ FW). Differences between 2017 and 2018 harvests were not significant. Malvidin‐3‐*O*‐glucoside (oenin) was the most prominent congener. About 15% of all anthocyanins were acetylated, and 15% were present as coumaric acid esters. ‘Cerason’, a variety from the Czech Republic, contained not only the highest total amounts of anthocyanins (5955.5 mg kg^−1^ FW) but also of acetyl esters (>30%). The dominating representative in the rosé‑ and white‐colored breeds was cyanidin‐3‐*O*‐glucoside (>97%).

**Figure 3:**
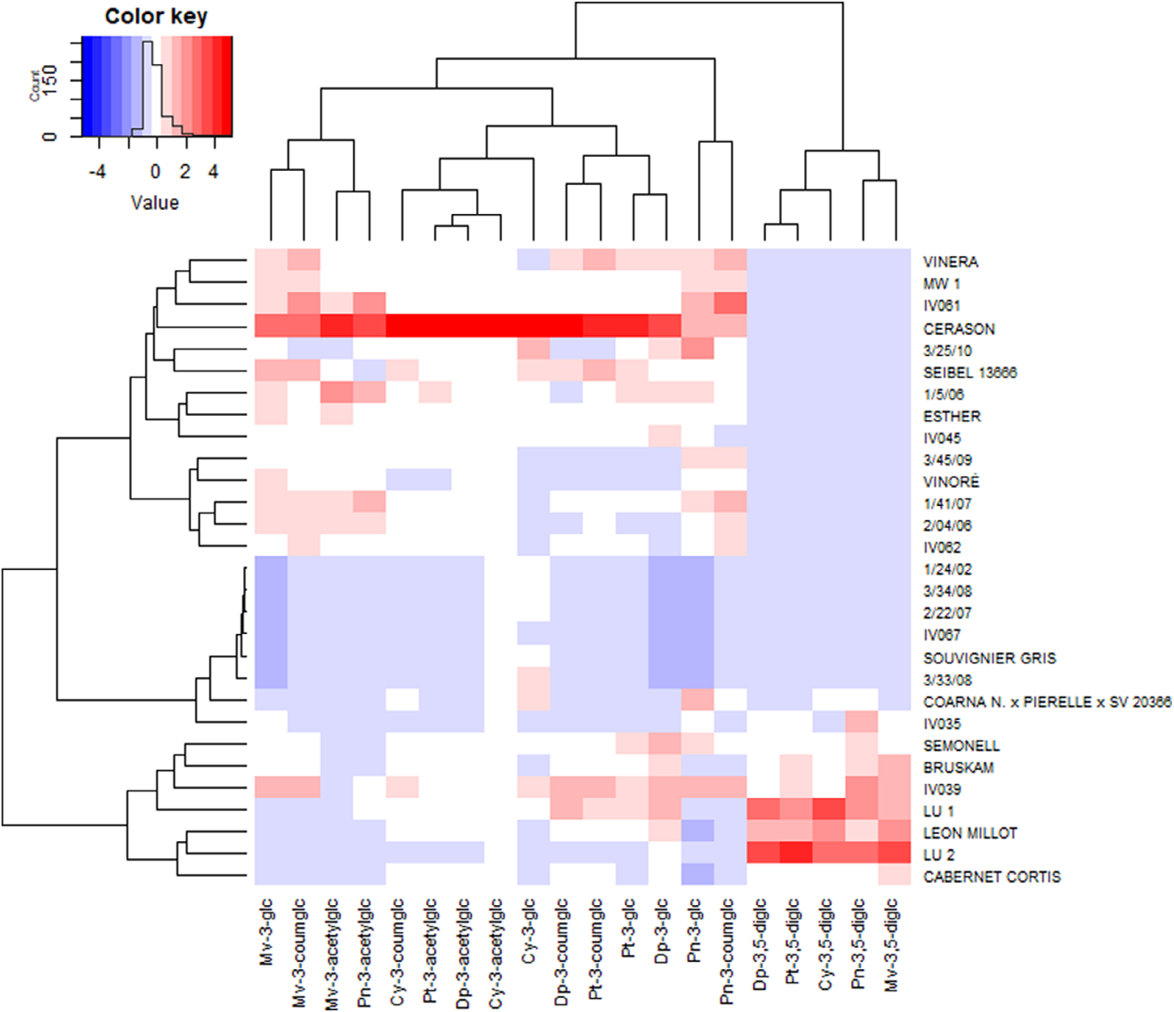
Heatmap of anthocyanin profiles in 29 grapevine hybrids in 2017 and 2018. Mv, malvidin; Dp, delphinidin; Cy, cyanidin; Pt, petunidin; Pn, peonidin; glc, glucose; diglc, diglucoside; acetylglc, acetylglucoside; coumglc, *p*‐coumaroylglucoside.

The determination of anthocyanin diglucosides, originating mainly from *Vitis* species other than *vinifera*, is of special importance for breeding programs since they are routinely used as potential biomarker to detect interspecific grapes or wines thereof. In this study, the 3,5‐*O*‐diglucosides were present in ten of the novel red BLs, but in none of the PIs (see Table 1). Cultivar ‘LU2’ was outstanding not only because of its highest content of diglucosides (average of 2295.5 mg kg^−1^ FW) but also because of their high proportion of about 90% of total anthocyanins in this plant. Further BLs with extraordinary high amounts were ‘LU1’ and ‘Leon Millot’ (45.3–61.6%). In ‘Coarna N. × Pierelle × SV 20366’ peonidin diglucoside was the most abundant derivative, whereas malvidin‐3,5‐*O* diglucoside was predominant in the other nine lines. Taking into account that the acceptable limit of diglucosides in wine is 15 mg L^−1^ (according to the International Organization of Vine and Wine^41^), five of the BLs with considerable amounts of malvidin‐3,5‐*O*‐diglucoside must be considered as problematic, particularly ‘LU1’, ‘LU2’, and ‘Leon Millot’, but also ‘Bruskam’ and ‘IV039’. An anthocyanins cluster analysis of the cultivars presented as a heatmap (see Fig. 3) showed clear clustering into two distinct groups, whereby the larger group is free of diglucosides. This cluster was further divided into those lines with higher amounts of anthocyanins and monoglucosides with or without acetyl and coumaroyl groups (‘Vinera’–‘IV062’) and a second group of those breeds with low amounts of anthocyanins and almost exclusively monoglucosides, particularly the rosé and white grapes (‘1/24/2’–‘IV035’).

### PIs *versus* BL

The 41 BLs of this study originated from several countries (Austria, Czech Republic, France, Germany, Hungary, Italy, Russia, Serbia, Switzerland, Ukraine, and Uzbekistan). The 17 new PIs were from a private breeder in South Tyrol (Supporting Information Table S1). All plants were grown in the same untreated vineyard. Hence, plant development variation due to different climatic conditions (such as rain quantity, temperature modulation, or sunshine duration) can be excluded. Therefore, any qualitative or quantitative differences in the polyphenol pattern should mirror differences in the genetic properties of the plants more exclusively than in other cultivation settings.

Comparing the results between the international BLs and the PIs that had never been investigated before, it became evident that the differences in the total amount of phenolics was insignificant. However, significant differences were found for several subclasses (see Supporting Information Table S9). The abundance of hydroxybenzoic acids was notably higher in the new PIs and was attributable to a threefold amount of gallic acid in white accessions (12.0 *versus* 4.2 mg kg^−1^ FW). White PIs also contained more flavonols than the white BLs did (epicatechin: 173.3 *versus* 75.3 mg kg^−1^ FW; epicatechin gallate: 71.1 *versus* 26.7 mg kg^−1^ FW; procyanidins B1–B4: 63.2 *versus* 37.9 mg kg^−1^ FW). The same tendency was observed for rutin and quercetin‐3‐*O*‐glucuronide (6.9 *versus* 4.2 mg kg^−1^ FW and 59.0 *versus* 29.5 mg kg^−1^ FW respectively). Luteolin‐7‐*O*‐glucoside was higher in white and red PIs. Anthocyanin diglucosides, particularly the legally problematic malvidin‐3,5‐*O*‐diglucoside, were not detectable in any of the PIs.

## CONCLUSION

The variations in the polyphenolic profiles of more than 50 different genotypes, obtained from either international breeding programs (BLs) or as absolutes (PIs) from private initiatives, were investigated. Variations between genotypes due to macroclimatic and pedologic conditions were excluded as all the grapevines were grown in one and the same untreated vineyard in northern Italy. This allowed the unbiased comparison and differentiation of the hybrids; differences found in the polyphenol pattern originate in the different genotypes of the hybrids solely. Variations of the polyphenolic profiles between two vintages were relatively limited, confirming the stability of the observed polyphenol patterns in the accessions investigated. Since this study was limited to single individuals, it is a must that these findings are reproduced in follow‐up studies in larger experimental cultivation plots. It was also observed that ten of the BLs investigated producing red‐colored grapes contained anthocyanin diglucosides. In nine of these the undesired malvidin‐3,5‐*O*‐diglucoside, a marker for non‐*vinifera* species, was even the most prominent diglucoside. In the context of the current EU legislation, half of these BLs must be considered as problematic. All new prodigy lines producing red grapes were found free of anthocyanin diglucosides. Summarizing, the knowledge gathered of the phenolic composition of grapes from all those breeding lines – most of them investigated for the first time – can serve as a fundament for further analytical studies, including ongoing breeding efforts towards further new highly resistant varieties with most desirable sensory characteristics.

## Supporting information


**Table S1.** List of studied accessions with names, internal (InnoVitis) code, VIVC (Vitis International Variety Catalogue) variety number, berry skin color, utilization, origin, agronomical and botanical information
**Table S2**. Soil parameters at growing site at Marlengo, South Tyrol, Italy.
**Table S3**. Concentration of phenolic classes in grape berries in mg kg‐1 FW (fresh weight) in harvest of 2017 and 2018
**Table S5**: Statistical analysis for repeated sampling (n = 3) in selected cultivars. Mean concentrations, standard deviations (SDs) and relative standard deviations (RSDs) of 20 key metabolites.
**Table S6**. Significance of variance for concentrations of phenolic classes between the harvest in 2017 and 2018
**Table S7**. Significance of variance for concentrations of phenolic classes between the red and white grape accessions
**Table S8**. Statistical significance of variance of concentrations of phenolic metabolites between 2017 and 2018 and between white and red grape accessions
**Table S9**. Concentrations of phenolic classes found in grapevine hybrids of different breeding origins harvested in 2017 and 2018 (in mg kg‐1 FW)
**Fig. S1**. PCA contribution plot of white grapes *vs*. average (anthocyanins excluded)
**Fig. S2**. PCA contribution plot of red grapes *vs*. average (anthocyanins excluded)
**Fig. S3**. Box plot illustrating the total phenolic content in all accessions (red and white grapes) in 2017 and 2018
**Fig. S4**. Box plot illustrating the total phenolic content in red (R) and white (W) accessions in 2017 and 2018
**Fig. S5**. Box plot illustrating the content of epicatechin in red (R) and white (W) accessions in 2017 and 2018
**Fig. S6**. Box plot illustrating the content of caftaric acid in red (R) and white (W) accessions in 2017 and 2018
**Fig. S7**. Heatmap of white grape accessions and identified compounds in the two harvests 2017 and 2018
**Fig. S8**. Heatmap of red grape accessions and identified compounds in the two harvests 2017 and 2018

**Table S4.** Concentration of phenolic compounds in grape berries in mg kg^−1^ FW (fresh weight) in harvest of 2017 and 2018 (provided as separate Excel file)
