# Supplementary material for: Comprehensive polyphenolic profiling in promising resistant grapevine hybrids including 17 novel breeds in northern Italy
Source: J Sci Food Agric. 2020 Oct 26;101(6):2380–8. doi: 10.1002/jsfa.10861 (PMC8048854; doi:10.1002/jsfa.10861)
Supplement: Supplementary file 1 — Table S1. List of studied accessions with names, internal (InnoVitis) code, VIVC (Vitis International Variety Catalogue) variety number, berry skin color, utilization, origin, agronomical and botanical information Table S2. Soil parameters at growing site at Marlengo, South Tyrol, Italy. Table S3. Concentration of phenolic classes in grape berries in mg kg‐1 FW (fresh weight) in harvest of 2017 and 2018 Table S5: Statistical analysis for repeated sampling (n = 3) in selected cultivars. Mean concentrations, standard deviations (SDs) and relative standard deviations (RSDs) of 20 key metabolites. Table S6. Significance of variance for concentrations of phenolic classes between the harvest in 2017 and 2018 Table S7. Significance of variance for concentrations of phenolic classes between the red and white grape accessions Table S8. Statistical significance of variance of concentrations of phenolic metabolites between 2017 and 2018 and between white and red grape accessions Table S9. Concentrations of phenolic classes found in grapevine hybrids of different breeding origins harvested in 2017 and 2018 (in mg kg‐1 FW) Fig. S1. PCA contribution plot of white grapes vs. average (anthocyanins excluded) Fig. S2. PCA contribution plot of red grapes vs. average (anthocyanins excluded) Fig. S3. Box plot illustrating the total phenolic content in all accessions (red and white grapes) in 2017 and 2018 Fig. S4. Box plot illustrating the total phenolic content in red (R) and white (W) accessions in 2017 and 2018 Fig. S5. Box plot illustrating the content of epicatechin in red (R) and white (W) accessions in 2017 and 2018 Fig. S6. Box plot illustrating the content of caftaric acid in red (R) and white (W) accessions in 2017 and 2018 Fig. S7. Heatmap of white grape accessions and identified compounds in the two harvests 2017 and 2018 Fig. S8. Heatmap of red grape accessions and identified compounds in the two harvests 2017 and 2018 [file JSFA-101-2380-s001.pdf]

Supporting information

Comprehensive polyphenolic profiling in promising resistant grapevine hybrids including seventeen novel breeds in Northern Italy

Running title: Phenolic profiling of fungus-resistant grapevine hybrids

Verena Gratl[a], Sonja Sturm[a*], Elena Zini[b], Thomas Letschka[b], Marco Stefanini[c], Silvia Vezzulli[c], and Hermann Stuppner[a]

[a] Institute of Pharmacy/Pharmacognosy, Center for Molecular Biosciences Innsbruck, University of Innsbruck, Innrain 80/82, Innsbruck 6020, Austria

[b] Laimburg Research Centre, Laimburg 6, 39050 Vadena (BZ), Italy

[c] Research and Innovation Centre, Fondazione Edmund Mach, via E. Mach 1, 38010 San Michele all'Adige (TN), Italy

Correspondence

Dr. Sonja Sturm, Institute of Pharmacy, CCB – Centrum of Chemistry and Biomedicine, University of Innsbruck, Department of Pharmacognosy, Innrain 80/82, 6020 Innsbruck, Austria. Phone: +4351250758408 Fax: +4351250758499 e-mail: sonja.sturm@uibk.ac.at

Description of supporting material:

Table S1. List of studied accessions with names, internal (InnoVitis) code, VIVC (Vitis International Variety Catalogue) variety number, berry skin color, utilization, origin, agronomical and botanical information

Table S2. Soil parameters at growing site at Marlengo, South Tyrol, Italy.

Table S3. Concentration of phenolic classes in grape berries in mg kg^{-1} FW (fresh weight) in harvest of 2017 and 2018

Table S4. Concentration of phenolic compounds in grape berries in mg kg^{-1} FW (fresh weight) in harvest of 2017 and 2018 (provided as separate Excel file)

Table S5: Statistical analysis for repeated sampling (n = 3) in selected cultivars. Mean concentrations, standard deviations (SDs) and relative standard deviations (RSDs) of twenty key metabolites.

Table S6. Significance of variance for concentrations of phenolic classes between the harvest in 2017 and 2018

Table S7. Significance of variance for concentrations of phenolic classes between the red and white grape accessions

Table S8. Statistical significance of variance of concentrations of phenolic metabolites between 2017 and 2018 and between white and red grape accessions

Table S9. Concentrations of phenolic classes found in grapevine hybrids of different breeding origins harvested in 2017 and 2018 (in mg kg^{-1} FW)

Figure S1. PCA contribution plot of white grapes versus average (anthocyanins excluded)

Figure S2. PCA contribution plot of red grapes versus average (anthocyanins excluded)

Figure S3. Box plot illustrating the total phenolic content in all accessions (red and white grapes) in 2017 and 2018

Figure S4. Box plot illustrating the total phenolic content in red (R) and white (W) accessions in 2017 and 2018

Figure S5. Box plot illustrating the content of epicatechin in red (R) and white (W) accessions in 2017 and 2018

Figure S6. Box plot illustrating the content of caftaric acid in red (R) and white (W) accessions in 2017 and 2018

Figure S7. Heatmap of white grape accessions and identified compounds in the two harvests 2017 and 2018

Figure S8. Heatmap of red grape accessions and identified compounds in the two harvests 2017 and 2018

Table S1. List of studied accessions with names, internal (InnoVitis) code, VIVC (Vitis International Variety Catalogue) variety number, berry skin color, utilization, origin, agronomical and botanical information[1,2]

Accession Name	InnoVitis code	Variety number VIVC	Berry skin color*	Utilization	Origin	Age of the vine	Yield	Species	Origin of mildew resistance
1/24/02	IV091	-	rose	wine grape	InnoVitis, Italy	2013	medium	Interspecific crossing	*V. rupestris*
1/26/4	IV132	-	blanc	wine grape	InnoVitis, Italy	2013	medium	Interspecific crossing	*V. rupestris*
1/41/07	IV104	-	noir	wine grape	InnoVitis, Italy	2013	medium	Interspecific crossing	*V. rupestris*
1/5/06	IV112	-	noir	wine grape	InnoVitis, Italy	2013	medium	Interspecific crossing	*V. rupestris/ V. lincecumii*
2/01/04	IV102	-	blanc	wine grape	InnoVitis, Italy	2013	medium	Interspecific crossing	*V.rupestris*
2/04/06	IV108	-	noir	wine grape	InnoVitis, Italy	2013	medium	Interspecific crossing	*V.rupestris*
2/22/07	IV078	-	rose	wine grape	InnoVitis, Italy	2013	medium	Interspecific crossing	*V.rupestris*
2/27/06	IV077	-	blanc	wine grape	InnoVitis, Italy	2013	medium	Interspecific crossing	*V. rupestris/ V. lincecumii*
2/31/10	IV098	-	blanc	wine grape	InnoVitis, Italy	2013	medium	Interspecific crossing	*V.rupestris*
3/1/06	IV076	-	blanc	wine grape	InnoVitis, Italy	2013	medium	Interspecific crossing	*V.rupestris*
3/14/10	IV096	-	blanc	wine grape	InnoVitis, Italy	2013	medium	Interspecific crossing	*V.rupestris*
3/23/05	IV075	-	blanc	wine grape	InnoVitis, Italy	2013	medium	Interspecific crossing	*V.rupestris*
3/25/10	IV083	-	noir	wine grape	InnoVitis, Italy	2013	medium	Interspecific crossing	*V.rupestris*
3/33/08	IV114	-	rose	wine grape	InnoVitis, Italy	2013	medium	Interspecific crossing	*V.rupestris*
3/34/08	IV107	-	rose	wine grape	InnoVitis, Italy	2013	medium	Interspecific crossing	*V.rupestris*
3/45/09	IV134	-	noir	wine grape	InnoVitis, Italy	2013	medium	Interspecific crossing	*V.rupestris*
4/5/08	IV110	-	blanc	wine grape	InnoVitis, Italy	2013	medium	Interspecific crossing	*V.rupestris*
(COARNA N. x PIERELLE) x SV 20366	IV037	-	noir	wine grape	Czech Republic	2010	medium	Interspecific crossing	*V.rupestris*
AROMERA	IV004	25400	blanc	wine grape	InnoVitis, Italy	2010	medium	*Vitis vinifera* Linné subsp. *vinifera*	*V. rupestris*
BRONNER	IV001	17129	blanc	wine grape	Germany	2010	medium	*Vitis vinifera* Linné subsp. *vinifera*	*V. labrusca or riparia/ V. amurensis*
BRUSKAM	IV038	16466	noir	wine grape	Russia	2010	medium	*Vitis* interspecific crossing	-
CABERNET CORTIS	IV051	20005	noir	wine grape	Germany	2010	medium	*Vitis vinifera* Linné subsp. *vinifera*	*V. labrusca or riparia/ V. amurensis*
CERASON	IV028	22821	noir	wine grape	Czech Republic	2010	medium	*Vitis* interspecific crossing	*V.rupestris*
DONAURIESLING	IV185	23901	blanc	wine grape	Austria	2010	medium	*Vitis* interspecific crossing	*V.rupestris*
DUNA GYÖNGYE	IV032	23137	blanc	wine grape	Hungary	2010	medium	*Vitis* interspecific crossing	*V.rupestris*
ESTHER	IV013	20341	noir	table grape	Hungary	2010	medium	*Vitis* interspecific crossing	*V.rupestris*
FANNY	IV011	20346	blanc	table grape	Hungary	2010	medium	*Vitis* interspecific crossing	*V.rupestris*
InnoVitis 029	IV029	-	blanc	wine grape	InnoVitis, Italy	2010	medium	Interspecific crossing	*V. amurensis*
InnoVitis 035	IV035	-	noir	wine grape	InnoVitis, Italy	2010	medium	Interspecific crossing	-
InnoVitis 039	IV039	-	noir	wine grape	InnoVitis, Italy	2010	medium	Interspecific crossing	*V. rupestris*
InnoVitis 043	IV043	-	blanc	wine grape	InnoVitis, Italy	2013	medium	Interspecific crossing	*M. rotundifolia/ V. amurensis*
InnoVitis 045	IV045	-	noir	wine grape	InnoVitis, Italy	2013	medium	Interspecific crossing	*M. rotundifolia/ V. amurensis*
InnoVitis 046	IV046	-	blanc	wine grape	InnoVitis, Italy	2010	medium	Interspecific crossing	*V.rupestris/ V. amurensis*
InnoVitis 061	IV061	-	noir	wine grape	InnoVitis, Italy	2013	high	Interspecific crossing	*M. rotundifolia/ V. amurensis*
InnoVitis 062	IV062	-	noir	wine grape	InnoVitis, Italy	2015	high	Interspecific crossing	*V.rupestris*
InnoVitis 063	IV063	-	blanc	wine grape	InnoVitis, Italy	2013	high	Interspecific crossing	*M. rotundifolia/ V. amurensis*
InnoVitis 064	IV064	-	blanc	wine grape	InnoVitis, Italy	2013	medium	Interspecific crossing	*M. rotundifolia/ V. amurensis*
InnoVitis 065	IV065	-	blanc	wine grape	InnoVitis, Italy	2013	high	Interspecific crossing	*M. rotundifolia/ V. amurensis*

Tabl S1. continued

Accession Name	InnoVitis code	Variety number VIVC	Berry skin color*	Utilization	Origin	Age of the vine	Yield	Species	Origin of mildew resistance
InnoVitis 066	IV066	-	blanc	wine grape	InnoVitis, Italy	2013	medium	Interspecific crossing	*M. rotundifolia/ V. amurensis*
InnoVitis 067	IV067	-	blanc	wine grape	InnoVitis, Italy	2013	high	Interspecific crossing	*M. rotundifolia/ V. amurensis*
LELA	IV187	14634	blanc	wine grape	Serbia	2010	medium	*Vitis* interspecific crossing	*V. amurensis*
LEON MILLOT	IV021	6806	noir	wine grape	France	2010	low	*Vitis* interspecific crossing	*V. riparia Michaux*
LIZA	IV188	14635	blanc	wine grape	Serbia	2010	medium	*Vitis* interspecific crossing	*V amurensis*
LU 1	IV040	-	noir	wine grape	Czech Republic	2010	medium	Interspecific crossing	*V. rupestris/ V. amurensis*
LU 2	IV036	-	noir	wine grape	Czech Republic	2010	medium	Interspecific crossing	*V. amurensis*
MW 1	IV027	-	noir	wine grape	Austria	2010	medium	Interspecific crossing	*V. rupestris/ V. lincecumii*
ODYSSEUS	IV190	17640	blanc	wine grape	Hungary	2010	medium	*Vitis* interspecific crossing	*V. amurensis*
PALATINA	IV009	14012	blanc	table grape	Hungary	2010	medium	*Vitis* interspecific crossing	*V. rupestris*
PETRA	IV195	14639	blanc	wine grape	Serbia	2010	medium	*Vitis* interspecific crossing	*V. amurensis*
PÖLÖSKEI MUSKOTALY	IV012	8207	blanc	table/wine grape	Hungary	2010	medium	*Vitis* interspecific crossing	*V.rupestris*
SEIBEL 13666	IV053	11408	noir	wine grape	France	2010	medium	*Vitis* interspecific crossing	*V.rupestris*
SEMONELL	IV041	-	noir	wine grape	Czech Republic	2010	medium	*Vitis* interspecific crossing	-
SEYVE VILLARD 12375	IV047	13081	blanc	wine grape	France	2010	medium	*Vitis* interspecific crossing	*V. rupestris*
SOLIRA	IV023	25402	blanc	wine grape	InnoVitis, Italy	2010	medium	*Vitis* interspecific crossing	*V. rupestris*
SOUVIGNIER GRIS	IV044	22629	blanc	wine grape	Germany	2010	medium	*Vitis vinifera* Linné subsp. *vinifera*	*V. rupestris/ V. lincecumii*
VICTORIA GYÖNGYE	IV031	14318	blanc	wine grape	Hungary	2010	medium	*Vitis* interspecific crossing	*V.rupestris*
VINERA	IV017	25404	noir	wine grape	InnoVitis, Italy	2010	high	*Vitis* interspecific crossing	*V. rupestris/ V. lincecumii*
VINORÈ	IV025	25403	noir	wine grape	InnoVitis, Italy	2010	medium	*Vitis* interspecific crossing	V.rupestris

* red grapes in the manuscript are grapes with berry skin color "noir", white grapes with color "blanc" according to Maul and Töpfer[2]

[1] Zini E, Dolzani C, Stefanini M, Gratl V, Bettinelli P, Nicolini D, Betta G, Dorigatti C, Velasco R, Letschka T and Vezzulli S. R -loci arrangement versus downy and powdery mildew resistance level: A Vitis hybrid survey.

[2] Maul E and Töpfer R. VIVC-Vitis International Variety Catalogue. Available: http://www.vivc.de/ [31 May 2019].

Table S2. Soil parameters at growing site at Marlengo, South Tyrol, Italy.

Parameter	
Soil type:	loamy sand (loam: 15 - 18%)
Thickness:	deep
Humus:	1.5 - 3%
pH:	6.5 - 6.7
Phosphor:	good
Potassium:	medium
Magnesium:	good
Iron:	good
Trace elements:	good
Calcium:	low

Table S3. Concentration of phenolic classes in grape berries in mg kg^{-1} FW (fresh weight) in harvest of 2017 and 2018

	2017							
Accession name	HBA	HCA	Stilbenes	Flavan-3-ols	Flavonols	Others	Anthocyanins	**Total**
1/24/02	23.36	91.45	6.94	710.39	132.71	3.37	42.89	**1011.10**
1/26/4	62.83	84.41	2.00	1767.81	143.29	4.65	n.d.	**2064.97**
1/41/07	19.64	54.71	5.06	844.13	126.97	4.09	1888.08	**2942.66**
1/5/06	22.36	32.21	1.10	461.36	127.28	3.39	2127.74	**2775.42**
2/01/04	37.18	76.44	1.91	1469.58	157.69	4.86	n.d.	**1747.64**
2/04/06	14.46	19.75	29.64	699.77	103.16	2.81	1393.80	**2263.38**
2/22/07	37.16	43.43	2.95	1145.14	192.28	5.06	47.45	**1473.46**
2/27/06	34.11	33.82	2.07	742.82	216.73	3.19	n.d.	**1032.73**
2/31/10	37.46	22.42	2.77	776.16	215.23	2.41	n.d.	**1056.44**
3/1/06	28.63	48.91	4.94	720.49	209.64	3.65	n.d.	**1016.25**
3/14/10	29.78	52.77	2.43	700.66	193.77	2.50	n.d.	**981.90**
3/23/05	33.52	30.04	5.84	781.56	178.34	3.56	n.d.	**1032.85**
3/25/10	18.74	39.87	2.39	638.66	95.94	3.54	1067.28	**1866.41**
3/33/08	25.42	10.82	0.47	774.16	164.99	3.40	72.63	**1051.88**
3/45/09	42.65	111.97	2.95	1167.79	184.96	5.39	818.77	**2334.46**
4/5/08	58.53	27.83	1.03	1042.56	121.83	3.67	n.d.	**1255.44**
3/34/08	21.83	39.01	8.30	580.21	93.41	3.02	40.82	**786.59**
COARNA N. x PIERELLE x SV 20366	15.76	24.19	7.68	303.55	127.48	3.61	508.70	**990.96**
IV046	21.07	52.81	4.25	836.93	133.00	3.47	n.d.	**1051.52**
AROMERA	7.38	13.27	2.16	289.08	57.32	1.40	n.d.	**370.59**
BRONNER	13.76	24.29	2.16	896.43	88.78	3.89	n.d.	**1029.30**
BRUSKAM	16.88	96.68	3.81	417.71	36.98	2.11	1810.95	**2385.11**
CABERNET CORTIS	27.40	101.31	4.92	1159.24	87.18	3.14	963.57	**2346.76**
CERASON	22.02	76.14	10.25	519.35	120.47	5.34	5762.81	**6516.37**
DONAURIESLING	27.69	76.91	1.11	779.87	95.35	4.69	n.d.	**985.60**
DUNA GYÖNGYE	11.52	28.35	5.16	417.18	48.47	3.58	n.d.	**514.25**
ESTHER	16.92	10.35	14.37	278.21	47.52	1.55	1378.60	**1747.51**
FANNY	10.11	5.31	10.62	178.83	36.27	0.77	n.d.	**241.90**
IV062	26.24	33.62	13.31	412.19	102.78	2.03	1062.29	**1652.44**
IV029	26.64	45.92	8.91	998.82	212.79	3.21	n.d.	**1296.27**
LELA	16.68	47.44	8.82	578.72	133.41	2.60	n.d.	**787.66**
LEON MILLOT	14.01	25.74	6.79	602.03	25.25	2.96	1428.78	**2105.55**
LIZA	14.58	29.82	0.68	254.73	105.58	3.33	n.d.	**408.70**
LU 1	8.60	15.66	4.54	605.61	13.27	3.56	2554.44	**3205.67**
LU 2	15.94	18.15	0.52	367.32	32.07	4.40	2759.56	**3197.95**
MW 1	22.55	22.64	36.96	577.05	93.23	3.26	1432.13	**2187.82**
ODYSSEUS	13.34	37.93	1.09	641.41	92.16	3.21	n.d.	**789.12**
PALATINA	17.53	18.72	1.61	796.46	81.95	2.24	n.d.	**918.49**
IV066	15.50	31.67	0.27	391.14	89.33	4.17	n.d.	**532.07**
IV043	16.62	55.66	3.00	467.33	145.68	3.16	n.d.	**691.44**
IV064	18.90	16.30	7.37	720.30	93.18	3.32	n.d.	**859.36**
IV065	14.93	4.30	74.86	348.02	196.59	3.75	n.d.	**642.44**
IV063	30.11	60.03	39.02	702.75	200.05	4.39	n.d.	**1036.34**
IV067	59.67	68.59	2.72	862.43	72.41	2.14	18.91	**1086.86**
IV061	5.57	23.89	12.43	351.39	67.69	2.90	1720.07	**2183.93**
IV045	21.09	19.92	11.03	364.56	134.50	1.44	1009.17	**1561.71**
PETRA	47.50	29.91	2.06	474.90	167.65	1.71	n.d.	**723.72**
PÖLÖSKEI MUSKOTALY	13.23	12.97	0.52	405.67	66.05	1.40	n.d.	**499.82**
SEIBEL 13666	19.35	84.56	13.35	658.32	77.94	4.31	1498.68	**2356.49**
SEMONELL	11.67	31.55	12.99	257.91	75.81	2.46	1877.63	**2270.02**
SEYVE VILLARD 12375	20.54	10.35	3.63	134.21	143.77	1.35	n.d.	**313.84**
SOLIRA	49.91	58.47	6.63	1116.77	199.14	4.05	n.d.	**1434.96**
SOUVIGNIER GRIS	9.93	14.02	0.97	203.63	87.36	2.12	44.14	**362.15**
IV039	45.57	47.29	30.26	605.10	79.41	2.55	2667.95	**3478.12**
IV035	27.20	66.24	1.97	274.96	58.95	3.07	993.27	**1425.66**
VIKTORIA GYÖNGYE	10.28	28.11	5.30	568.49	81.87	1.19	n.d.	**695.23**
VINERA	15.85	9.69	25.05	262.40	40.10	1.06	1817.73	**2171.87**
VINORÈ	18.40	24.02	3.13	1019.50	46.94	1.51	1041.50	**2154.98**

	2018							
Accession name	HBA	HCA	Stilbenes	Flavan-3-ols	Flavonols	Others	Anthocyanins	**Total**
1/24/02	19.02	86.41	1.95	905.23	74.57	3.55	29.3	**1120.06**
1/26/4	42.68	55.79	3.62	723.62	127.15	2.48	n.d.	**955.33**
1/41/07	17.20	46.11	8.48	929.26	44.03	4.45	1048.8	**2098.35**
1/5/06	13.80	36.65	3.32	389.71	57.36	2.93	1554.0	**2057.79**
2/01/04	30.78	45.24	1.33	649.50	136.88	4.31	n.d.	**868.02**
2/04/06	12.36	25.41	23.45	498.63	46.50	2.23	931.9	**1540.48**
2/22/07	44.58	36.49	0.76	1132.10	116.74	4.99	33.2	**1368.83**
2/27/06	38.67	55.85	0.75	782.43	227.62	5.75	n.d.	**1111.06**
2/31/10	35.73	70.94	3.66	1161.25	117.82	3.52	n.d.	**1392.90**
3/1/06	41.53	67.77	11.32	688.69	263.36	4.42	n.d.	**1077.07**
3/14/10	44.69	74.48	3.34	1057.58	196.59	4.29	n.d.	**1380.96**
3/23/05	36.93	41.61	8.63	726.28	141.84	4.19	n.d.	**959.48**
3/25/10	21.12	38.05	1.30	544.01	80.48	3.05	713.4	**1401.37**
3/33/08	31.98	13.39	1.95	840.57	67.75	2.21	84.3	**1042.19**
3/45/09	45.52	120.19	14.23	840.61	107.76	4.46	731.1	**1863.85**
4/5/08	44.78	31.16	3.27	816.93	166.80	3.36	n.d.	**1066.29**
3/34/08	18.55	25.73	9.45	564.06	55.11	3.42	34.0	**710.33**
COARNA N. x PIERELLE x SV 20366	12.36	20.73	5.31	339.96	93.86	3.83	269.7	**745.70**
IV046	30.20	60.82	5.36	938.01	211.05	3.99	n.d.	**1249.41**
AROMERA	29.86	36.40	0.27	943.17	122.69	2.52	n.d.	**1134.89**
BRONNER	23.63	60.63	0.36	583.94	143.68	4.19	n.d.	**816.41**
BRUSKAM	28.60	99.38	1.26	438.70	43.82	3.72	1397.8	**2013.29**
CABERNET CORTIS	15.91	118.67	2.12	778.68	70.23	4.36	772.6	**1762.57**
CERASON	14.47	191.55	47.91	584.66	82.94	6.48	6148.1	**7076.11**
DONAURIESLING	21.57	123.87	3.72	711.63	86.65	3.82	n.d.	**951.25**
DUNA GYÖNGYE	15.79	60.60	5.81	501.91	100.99	3.11	n.d.	**688.20**
ESTHER	11.16	29.14	6.49	123.47	35.90	1.07	1212.9	**1420.10**
FANNY	3.73	12.94	8.46	146.07	9.95	1.10	n.d.	**182.24**
IV062	31.58	40.01	2.32	503.80	145.24	2.58	1059.2	**1784.73**
IV029	31.16	49.24	15.73	1291.73	336.01	4.56	n.d.	**1728.41**
LELA	18.64	77.44	4.73	379.57	137.71	2.06	n.d.	**620.14**
LEON MILLOT	36.32	74.45	18.72	554.10	31.07	3.37	2346.8	**3064.84**
LIZA	16.10	48.53	1.16	301.81	77.84	3.23	n.d.	**448.66**
LU 1	12.71	15.68	6.32	483.34	29.76	4.12	2374.9	**2926.77**
LU 2	12.49	19.95	2.99	287.90	37.05	3.03	2450.8	**2814.22**
MW 1	28.30	32.06	28.29	690.59	149.19	3.86	1326.5	**2258.76**
ODYSSEUS	15.66	72.43	0.71	1162.41	63.07	3.71	n.d.	**1317.98**
PALATINA	17.89	23.63	5.49	57.03	91.19	0.84	n.d.	**196.07**
IV066	17.05	58.38	1.09	320.95	68.48	3.01	n.d.	**468.94**
IV043	15.92	53.78	1.55	595.74	101.88	3.04	n.d.	**771.89**
IV064	33.46	63.25	4.52	923.22	141.66	3.76	n.d.	**1169.85**
IV065	37.08	18.45	5.17	952.41	126.99	3.72	n.d.	**1143.80**
IV063	34.25	100.71	99.65	980.98	254.26	6.09	n.d.	**1475.92**
IV067	75.61	58.82	34.01	407.64	95.34	1.92	30.8	**704.07**
IV061	11.46	35.04	18.15	250.30	36.74	1.86	1734.2	**2087.72**
IV045	24.50	37.69	1.72	455.36	84.94	2.02	1258.7	**1864.94**
PETRA	49.87	99.81	0.94	363.52	194.17	2.47	n.d.	**710.76**
PÖLÖSKEI MUSKOTALY	13.21	9.47	2.41	203.98	78.09	1.20	n.d.	**308.34**
SEIBEL 13666	24.75	61.11	38.84	648.43	130.57	2.45	2131.4	**3037.53**
SEMONELL	22.24	30.90	1.66	256.18	69.47	2.40	1140.1	**1522.94**
SEYVE VILLARD 12375	21.86	18.98	8.95	198.69	115.40	2.02	n.d.	**365.89**
SOLIRA	47.14	77.99	2.27	1126.51	169.31	3.59	n.d.	**1426.80**
SOUVIGNIER GRIS	11.98	41.20	0.71	84.99	128.47	2.55	46.9	**316.80**
IV039	24.39	70.47	23.71	448.49	58.50	2.77	2700.2	**3328.48**
IV035	12.11	74.42	3.55	427.80	63.16	3.66	876.6	**1461.29**
VIKTORIA GYÖNGYE	23.55	41.84	4.80	559.32	149.91	1.05	n.d.	**780.46**
VINERA	28.51	19.89	24.34	246.77	43.43	2.18	1678.3	**2043.38**
VINORÈ	57.14	43.06	3.11	1344.75	70.71	2.88	670.3	**2191.90**

HBA….*p*-Hydroxybenzoic acids, HCA…Hydroxycinnamic acids

Table S5a: Statistical analysis for repeated sampling (n = 3) in selected cultivars (n = 8). Mean concentrations, standard deviations (SDs) and relative standard deviations (RSDs) for twenty key polyphenolic metabolites.

Polyphenols (mg/kg FW)<br>Accession		gallic acid	ellagic acid	caftaric acid	fertaric acid	t-resveratrol	t-piceide	cis-piceide	catechin	epicatechin	gallocatechin	epicatechin gallate	procyanidin B1	procyanidin B2 + B4	procyanidin B3 (as B1)	quercetin-3-glc / -3-gal	isorhamnetin-3-Glc	quercetin-3-glu	kaempferol-3-glu	luteolin-7-O-Glc	kaempferol-3-Glc
LIZA	sample 1	0.92	13.16	26.71	3.11	n.d.	0.23	0.46	110.20	34.35	1.95	15.09	56.89	30.46	5.80	57.22	0.50	33.73	0.78	2.41	5.13
	sample 2	1.22	13.69	31.13	3.93	n.d.	0.23	0.63	83.50	56.40	2.40	17.44	57.15	33.41	7.78	60.06	0.94	38.47	0.93	2.53	4.86
	sample 3	0.96	10.11	30.75	3.52	n.d.	0.27	0.82	110.35	43.00	1.84	16.44	56.45	27.52	5.77	50.79	0.43	35.00	0.53	1.82	3.98
	**Mean**	**1.03**	**12.32**	**29.53**	**3.52**		**0.24**	**0.64**	**101.35**	**44.58**	**2.06**	**16.32**	**56.83**	**30.46**	**6.45**	**56.02**	**0.62**	**35.73**	**0.74**	**2.25**	**4.66**
	**SD**	**0.16**	**1.93**	**2.45**	**0.41**		**0.02**	**0.18**	**15.46**	**11.11**	**0.30**	**1.18**	**0.35**	**2.95**	**1.15**	**4.75**	**0.28**	**2.45**	**0.20**	**0.38**	**0.60**
	**RSD (%)**	**15.76**	**15.67**	**8.29**	**11.66**		**10.21**	**28.75**	**15.25**	**24.92**	**14.41**	**7.22**	**0.62**	**9.67**	**17.85**	**8.48**	**45.05**	**6.86**	**27.24**	**16.90**	**12.88**
2/27/06	sample 1	3.00	29.62	30.79	3.03	0.17	0.24	1.58	438.40	136.75	1.28	34.15	45.13	62.74	24.38	111.67	3.96	60.25	1.96	2.95	8.04
	sample 2	4.56	25.51	23.42	3.33	0.11	0.60	1.78	447.35	163.65	1.51	41.33	63.18	62.36	24.46	116.74	5.59	61.00	2.48	2.73	9.84
	sample 3	3.55	22.29	30.12	3.62	0.17	0.99	1.67	522.10	159.05	1.33	40.66	72.54	64.14	36.91	125.96	6.26	62.64	2.22	3.29	10.38
	**Mean**	**3.70**	**25.81**	**28.11**	**3.32**	**0.15**	**0.61**	**1.67**	**469.28**	**153.15**	**1.37**	**38.71**	**60.28**	**63.08**	**28.58**	**118.12**	**5.27**	**61.29**	**2.22**	**2.99**	**9.42**
	**SD**	**0.79**	**3.67**	**4.08**	**0.30**	**0.04**	**0.37**	**0.10**	**45.96**	**14.39**	**0.12**	**3.97**	**13.93**	**0.94**	**7.21**	**7.24**	**1.18**	**1.22**	**0.26**	**0.28**	**1.22**
	**RSD (%)**	**21.37**	**14.23**	**14.50**	**8.95**	**24.66**	**61.42**	**6.14**	**9.79**	**9.39**	**8.89**	**10.25**	**23.11**	**1.49**	**25.23**	**6.13**	**22.44**	**1.99**	**11.73**	**9.54**	**13.00**
DUNA GYÖNGYE	sample 1	4.06	7.00	25.73	2.62	0.23	1.17	3.77	203.60	78.50	7.57	26.64	45.08	37.92	17.88	29.36	0.10	14.25	0.27	1.81	1.04
	sample 2	3.21	4.41	28.92	3.03	0.24	3.02	7.58	227.85	46.95	2.69	9.81	41.91	39.76	14.71	38.88	0.16	10.74	0.16	1.04	0.36
	sample 3	1.98	9.93	24.97	4.18	0.43	4.39	7.94	285.60	77.95	3.40	13.86	57.65	38.07	14.74	63.71	0.24	19.92	0.24	1.00	1.09
	**Mean**	**3.08**	**7.11**	**26.54**	**3.27**	**0.30**	**2.86**	**6.43**	**239.02**	**67.80**	**4.55**	**16.77**	**48.21**	**38.58**	**15.77**	**43.98**	**0.16**	**14.97**	**0.22**	**1.28**	**0.83**
	**SD**	**1.05**	**2.76**	**2.10**	**0.81**	**0.11**	**1.62**	**2.31**	**42.13**	**18.06**	**2.63**	**8.79**	**8.33**	**1.02**	**1.82**	**17.73**	**0.07**	**4.63**	**0.05**	**0.46**	**0.41**
	**RSD (%)**	**33.93**	**38.81**	**7.90**	**24.79**	**37.47**	**56.58**	**35.97**	**17.62**	**26.64**	**57.86**	**52.40**	**17.27**	**2.64**	**11.55**	**40.32**	**44.61**	**30.95**	**24.74**	**35.73**	**49.08**
FANNY	sample 1	4.48	5.44	4.81	0.50	0.26	3.00	7.36	104.30	41.20	0.79	6.30	7.15	16.00	3.11	4.02	2.48	4.67	0.10	0.72	n.d.
	sample 2	3.22	4.09	4.44	0.44	0.15	2.44	9.13	110.70	43.30	0.20	5.57	7.25	15.49	2.95	7.08	0.72	4.54	0.07	0.74	n.d.
	sample 3	2.61	3.08	5.70	0.55	0.27	1.96	4.97	63.90	19.45	1.10	3.61	4.90	13.18	3.32	4.96	0.39	3.62	0.04	0.59	n.d.
	**Mean**	**3.43**	**4.20**	**4.98**	**0.50**	**0.22**	**2.47**	**7.15**	**92.97**	**34.65**	**0.69**	**5.16**	**6.43**	**14.89**	**3.12**	**5.35**	**1.20**	**4.28**	**0.07**	**0.68**	
	**SD**	**0.95**	**1.18**	**0.65**	**0.05**	**0.07**	**0.52**	**2.09**	**25.38**	**13.21**	**0.45**	**1.39**	**1.33**	**1.50**	**0.18**	**1.57**	**1.13**	**0.58**	**0.03**	**0.08**	
	**RSD (%)**	**27.71**	**28.14**	**13.03**	**10.64**	**30.40**	**21.01**	**29.23**	**27.29**	**38.11**	**65.55**	**26.95**	**20.69**	**10.08**	**5.87**	**29.30**	**94.17**	**13.46**	**44.11**	**12.24**	
3/23/05	sample 1	14.47	17.84	25.44	4.60	0.61	1.97	2.98	246.90	281.25	4.63	72.31	61.19	77.37	37.17	87.94	3.78	57.80	1.24	2.73	5.44
	sample 2	18.18	19.11	21.83	3.93	1.31	1.73	3.68	303.80	307.80	7.81	95.80	71.80	89.61	48.75	69.20	4.31	64.79	0.88	3.57	3.82
	sample 3	16.20	16.30	27.55	3.25	1.17	1.45	2.05	341.15	253.50	6.14	102.86	74.24	106.22	37.96	46.48	2.84	55.13	0.47	3.16	1.90
	**Mean**	**16.28**	**17.75**	**24.94**	**3.92**	**1.03**	**1.72**	**2.90**	**297.28**	**280.85**	**6.19**	**90.32**	**69.07**	**91.06**	**41.29**	**67.87**	**3.64**	**59.24**	**0.86**	**3.15**	**3.72**
	**SD**	**1.86**	**1.41**	**2.89**	**0.68**	**0.37**	**0.26**	**0.82**	**47.46**	**27.15**	**1.59**	**15.99**	**6.94**	**14.48**	**6.47**	**20.76**	**0.74**	**4.99**	**0.39**	**0.42**	**1.77**
	**RSD (%)**	**11.40**	**7.94**	**11.60**	**17.21**	**36.14**	**15.17**	**28.11**	**15.97**	**9.67**	**25.66**	**17.71**	**10.04**	**15.90**	**15.67**	**30.59**	**20.43**	**8.42**	**44.63**	**13.25**	**47.75**
2/04/06	sample 1	3.42	9.94	17.25	2.51	1.81	6.23	19.39	284.55	246.75	1.74	14.28	42.46	90.85	19.14	56.81	6.45	28.00	0.40	1.93	2.52
	sample 2	3.52	7.81	18.16	2.69	1.53	6.01	11.48	284.60	204.30	3.02	14.08	51.34	92.54	19.53	67.50	6.18	33.82	0.34	1.94	3.37
	sample 3	2.24	12.25	25.81	3.11	4.27	7.65	19.46	243.15	185.30	3.04	21.18	46.90	81.04	19.28	47.19	5.87	30.36	0.24	2.19	2.27
	**Mean**	**3.06**	**10.00**	**20.41**	**2.77**	**2.53**	**6.63**	**16.78**	**270.77**	**212.12**	**2.60**	**16.51**	**46.90**	**88.14**	**19.32**	**57.17**	**6.16**	**30.73**	**0.33**	**2.02**	**2.72**
	**SD**	**0.71**	**2.22**	**4.70**	**0.31**	**1.51**	**0.89**	**4.59**	**23.92**	**31.46**	**0.74**	**4.04**	**4.44**	**6.21**	**0.19**	**10.16**	**0.29**	**2.93**	**0.08**	**0.15**	**0.58**
	**RSD (%)**	**23.22**	**22.23**	**23.05**	**11.23**	**59.55**	**13.42**	**27.34**	**8.83**	**14.83**	**28.61**	**24.49**	**9.47**	**7.05**	**1.01**	**17.77**	**4.71**	**9.53**	**24.74**	**7.37**	**21.24**
COARNA N. x PIERELLE x SV 20366	sample 1	1.87	13.04	19.61	4.58	1.44	2.74	4.17	106.30	54.95	18.53	12.92	66.05	29.42	14.95	75.41	3.73	32.36	0.66	1.95	2.73
	sample 2	1.64	17.79	12.53	5.27	1.19	3.42	3.48	175.85	93.00	19.42	23.09	100.63	54.24	29.62	104.77	6.05	49.94	1.20	2.86	4.77
	sample 3	2.55	15.07	15.28	6.01	2.83	4.13	6.27	149.40	53.30	22.43	22.53	110.73	49.21	21.80	84.17	4.76	48.11	1.03	2.68	3.11
	**Mean**	**2.02**	**15.30**	**15.81**	**5.28**	**1.82**	**3.43**	**4.64**	**143.85**	**67.08**	**20.13**	**19.51**	**92.47**	**44.29**	**22.12**	**88.12**	**4.85**	**43.47**	**0.96**	**2.50**	**3.54**
	**SD**	**0.47**	**2.38**	**3.57**	**0.72**	**0.89**	**0.70**	**1.45**	**35.11**	**22.46**	**2.04**	**5.72**	**23.43**	**13.12**	**7.34**	**15.07**	**1.16**	**9.67**	**0.27**	**0.48**	**1.09**
	**RSD (%)**	**23.48**	**15.56**	**22.61**	**13.58**	**48.79**	**20.29**	**31.35**	**24.40**	**33.48**	**10.15**	**29.29**	**25.34**	**29.62**	**33.20**	**17.11**	**23.94**	**22.24**	**28.47**	**19.28**	**30.74**
MW 1	sample 1	4.47	17.45	20.41	2.23	7.21	8.50	18.87	242.30	194.55	3.71	28.32	29.00	62.48	16.45	38.91	1.89	43.20	0.16	2.19	0.67
	sample 2	5.98	17.92	23.29	2.53	11.84	10.52	28.11	303.70	191.00	4.49	36.19	41.60	92.77	20.92	45.18	1.85	51.48	0.44	2.16	0.72
	sample 3	5.42	11.48	20.22	1.89	8.24	10.07	23.01	229.35	146.20	3.00	23.01	29.40	62.14	10.99	41.10	1.73	40.92	0.10	1.91	0.62
	**Mean**	**5.29**	**15.62**	**21.31**	**2.22**	**9.09**	**9.70**	**23.33**	**258.45**	**177.25**	**3.73**	**29.17**	**33.33**	**72.46**	**16.12**	**41.73**	**1.82**	**45.20**	**0.23**	**2.08**	**0.67**
	**SD**	**0.77**	**3.59**	**1.72**	**0.32**	**2.43**	**1.06**	**4.63**	**39.72**	**26.95**	**0.75**	**6.63**	**7.16**	**17.58**	**4.98**	**3.18**	**0.08**	**5.56**	**0.18**	**0.16**	**0.05**
	**RSD (%)**	**14.49**	**22.98**	**8.07**	**14.57**	**26.76**	**10.94**	**19.84**	**15.37**	**15.20**	**19.99**	**22.74**	**21.48**	**24.27**	**30.87**	**7.63**	**4.57**	**12.29**	**78.11**	**7.46**	**7.88**

Table S5a: Statistical analysis for repeated sampling (n = 3) in selected cultivars (n = 8). Mean concentrations, standard deviations (SDs) and relative standard deviations (RSDs) for twenty key polyphenolic metabolites.

Accession	LIZA			2/27/06			DUNA GYÖNGYE			FANNY			3/23/05			2/04/06			COARNA N. x PIERELLE x SV 20366			MW 1		
all data mgkg $^{-1}$	Mean	SD	RSD (%)	Mean	SD	RSD (%)	Mean	SD	RSD (%)	Mean	SD	RSD (%)	Mean	SD	RSD (%)	Mean	SD	RSD (%)	Mean	SD	RSD (%)	MW	SD	RSD (%)
gallic acid	1.03	0.16	15.76	3.70	0.79	21.37	3.08	1.05	33.93	3.43	0.95	27.71	16.28	1.86	11.40	3.06	0.71	23.22	2.02	0.47	23.48	5.29	0.77	14.49
ellagic acid	12.32	1.93	15.67	25.81	3.67	14.23	7.11	2.76	38.81	4.20	1.18	28.14	17.75	1.41	7.94	10.00	2.22	22.23	15.30	2.38	15.56	15.62	3.59	22.98
caftaric acid	29.53	2.45	8.29	28.11	4.08	14.50	26.54	2.10	7.90	4.98	0.65	13.03	24.94	2.89	11.60	20.41	4.70	23.05	15.81	3.57	22.61	21.31	1.72	8.07
fertaric acid	3.52	0.41	11.66	3.32	0.30	8.95	3.27	0.81	24.79	0.50	0.05	10.64	3.92	0.68	17.21	2.77	0.31	11.23	5.28	0.72	13.58	2.22	0.32	14.57
t-resveratrol				0.15	0.04	24.66	0.30	0.11	37.47	0.22	0.07	30.40	1.03	0.37	36.14	2.53	1.51	59.55	1.82	0.89	48.79	9.09	2.43	26.76
t-piceide	0.24	0.02	10.21	0.61	0.37	61.42	2.86	1.62	56.58	2.47	0.52	21.01	1.72	0.26	15.17	6.63	0.89	13.42	3.43	0.70	20.29	9.70	1.06	10.94
cis-piceide	0.64	0.18	28.75	1.67	0.10	6.14	6.43	2.31	35.97	7.15	2.09	29.23	2.90	0.82	28.11	16.78	4.59	27.34	4.64	1.45	31.35	23.33	4.63	19.84
catechin	101.35	15.46	15.25	469.28	45.96	9.79	239.02	42.13	17.62	92.97	25.38	27.29	297.28	47.46	15.97	270.77	23.92	8.83	143.85	35.11	24.40	258.45	39.72	15.37
epicatechin	44.58	11.11	24.92	153.15	14.39	9.39	67.80	18.06	26.64	34.65	13.21	38.11	280.85	27.15	9.67	212.12	31.46	14.83	67.08	22.46	33.48	177.25	26.95	15.20
gallocatechin	2.06	0.30	14.41	1.37	0.12	8.89	4.55	2.63	57.86	0.69	0.45	65.55	6.19	1.59	25.66	2.60	0.74	28.61	20.13	2.04	10.15	3.73	0.75	19.99
epicatechin gallate	16.32	1.18	7.22	38.71	3.97	10.25	16.77	8.79	52.40	5.16	1.39	26.95	90.32	15.99	17.71	16.51	4.04	24.49	19.51	5.72	29.29	29.17	6.63	22.74
procyanidin B1	56.83	0.35	0.62	60.28	13.93	23.11	48.21	8.33	17.27	6.43	1.33	20.69	69.07	6.94	10.04	46.90	4.44	9.47	92.47	23.43	25.34	33.33	7.16	21.48
procyanidin B2 + B4	30.46	2.95	9.67	63.08	0.94	1.49	38.58	1.02	2.64	14.89	1.50	10.08	91.06	14.48	15.90	88.14	6.21	7.05	44.29	13.12	29.62	72.46	17.58	24.27
procyanidin B3 (as B1)	6.45	1.15	17.85	28.58	7.21	25.23	15.77	1.82	11.55	3.12	0.18	5.87	41.29	6.47	15.67	19.32	0.19	1.01	22.12	7.34	33.20	16.12	4.98	30.87
quercetin-3-glc / -3-gal	56.02	4.75	8.48	118.12	7.24	6.13	43.98	17.73	40.32	5.35	1.57	29.30	67.87	20.76	30.59	57.17	10.16	17.77	88.12	15.07	17.11	41.73	3.18	7.63
isorhamnetin-3-Glc	0.62	0.28	45.05	5.27	1.18	22.44	0.16	0.07	44.61	1.20	1.13	94.17	3.64	0.74	20.43	6.16	0.29	4.71	4.85	1.16	23.94	1.82	0.08	4.57
quercetin-3-glu	35.73	2.45	6.86	61.29	1.22	1.99	14.97	4.63	30.95	4.28	0.58	13.46	59.24	4.99	8.42	30.73	2.93	9.53	43.47	9.67	22.24	45.20	5.56	12.29
kaempferol-3-glu	0.74	0.20	27.24	2.22	0.26	11.73	0.22	0.05	24.74	0.07	0.03	44.11	0.86	0.39	44.63	0.33	0.08	24.74	0.96	0.27	28.47	0.23	0.18	78.11
luteolin-7-O-Glc	2.25	0.38	16.90	2.99	0.28	9.54	1.28	0.46	35.73	0.68	0.08	12.24	3.15	0.42	13.25	2.02	0.15	7.37	2.50	0.48	19.28	2.08	0.16	7.46
kaempferol-3-Glc	4.66	0.60	12.88	9.42	1.22	13.00	0.83	0.41	49.08				3.72	1.77	47.75	2.72	0.58	21.24	3.54	1.09	30.74	0.67	0.05	7.88

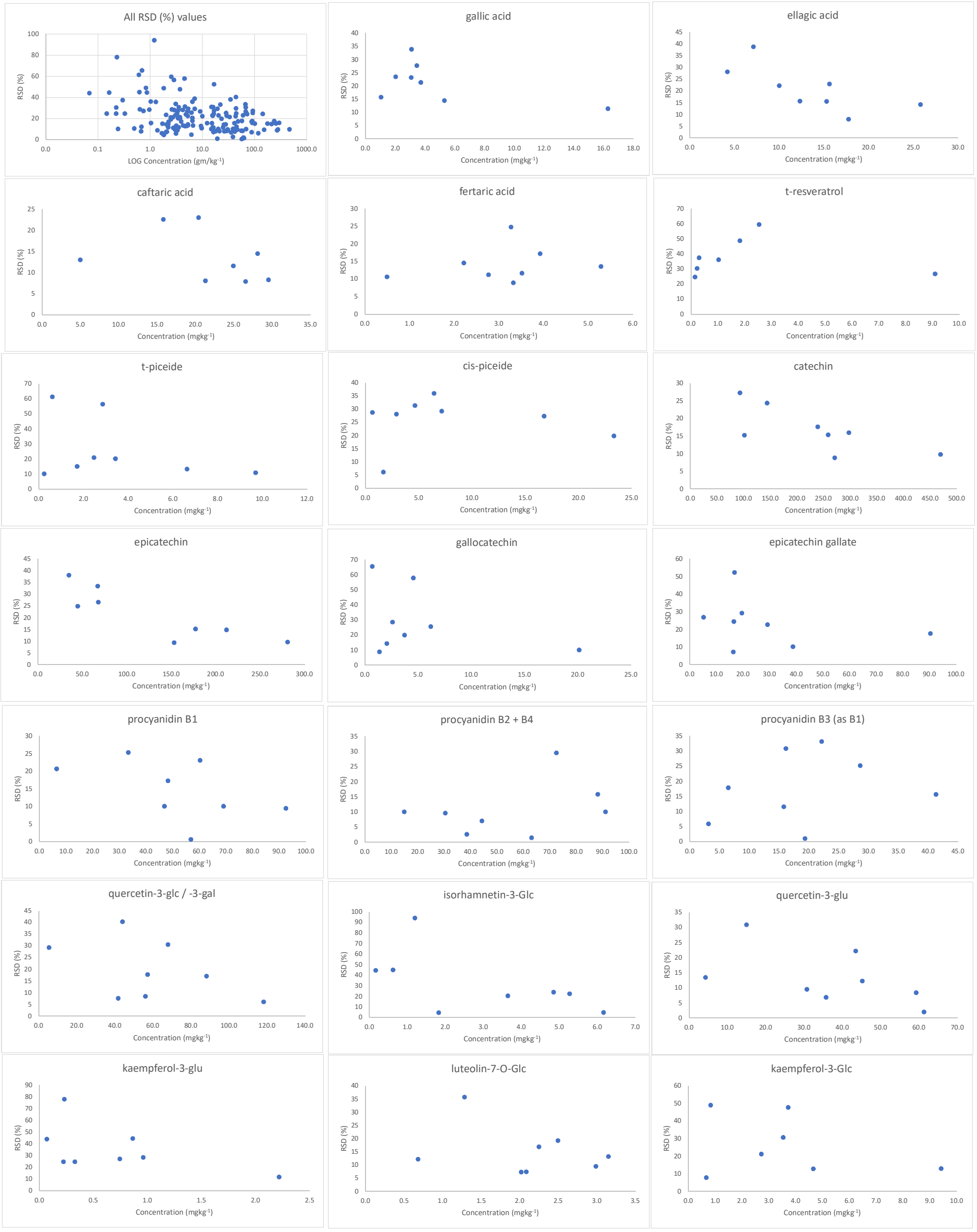

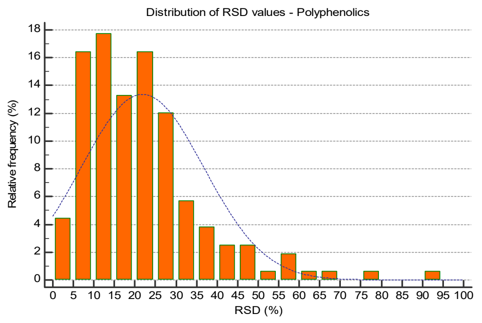

Table S5b: Statistical analysis for repeated sampling (n = 3) in selected cultivars (n = 3). Mean concentrations, standard deviations (SDs) and relative standard deviations (RSDs) for twenty polyphenolic key metabolites of the anthocyanin structure class.

Polyphenols (mg/kg FW)<br>Accession		Dp 3-glc	Cn 3-glc	Pt 3-glc	Pn 3-glc	Mv 3-glc	Dp acilata	Cn acilata	Pt acilata	Pn acilata	Mv acilata	Dp p-cumarata	Cn p-cumarata	Pt p-cumarata	Pn p-cumarata	Mv p-cumarata	MV 3,5-diglucoside	DP 3,5-diglucoside	CN 3,5-diglucoside	PT 3,5-diglucoside	PN 3,5-diglucoside
2/04/06	sample 1	53.11	14.45	95.31	69.35	556.35	11.32	3.52	26.64	29.17	242.20	11.60	9.01	23.74	30.06	218.01	n.d.	n.d.	n.d.	n.d.	n.d.
	sample 2	43.31	12.09	77.83	65.78	521.30	9.33	2.71	22.26	29.36	222.85	10.97	8.26	22.27	29.32	222.37	n.d.	n.d.	n.d.	n.d.	n.d.
	sample 3	49.29	11.42	88.43	62.52	593.00	12.19	3.15	26.64	29.24	300.70	11.16	8.88	23.79	32.67	228.87	n.d.	n.d.	n.d.	n.d.	n.d.
	**Mean**	**48.57**	**12.65**	**87.19**	**65.88**	**556.88**	**10.94**	**3.13**	**25.18**	**29.25**	**255.25**	**11.24**	**8.71**	**23.26**	**30.68**	**223.08**					
	**SD**	**4.04**	**1.30**	**7.19**	**2.79**	**29.27**	**1.20**	**0.33**	**2.07**	**0.08**	**33.09**	**0.26**	**0.33**	**0.70**	**1.44**	**4.46**					
	**RSD (%)**	**8.31**	**10.28**	**8.24**	**4.23**	**5.26**	**10.94**	**10.59**	**8.21**	**0.28**	**12.97**	**2.33**	**3.77**	**3.02**	**4.68**	**2.00**					
COARNA N. x PIERELLE x SV 20366	sample 1	49.26	106.23	57.11	135.08	84.43	0.22	0.87	0.36	1.66	1.01	2.58	9.59	1.76	13.53	10.57	8.38	n.d.	2.35	3.00	20.75
	sample 2	35.69	103.86	38.60	106.12	53.82	0.14	0.78	0.21	1.39	0.88	1.50	7.34	0.92	9.81	6.52	4.73	n.d.	2.24	1.94	13.74
	sample 3	46.71	113.19	47.62	123.76	58.59	0.68	1.33	0.68	1.62	1.31	2.80	8.94	1.61	10.84	7.76	5.09	n.d.	2.46	2.33	14.86
	**Mean**	**43.89**	**107.76**	**47.78**	**121.65**	**65.61**	**0.35**	**0.99**	**0.42**	**1.55**	**1.07**	**2.29**	**8.62**	**1.43**	**11.39**	**8.28**	**6.07**		**2.35**	**2.42**	**16.45**
	**SD**	**5.89**	**3.96**	**7.56**	**11.91**	**13.45**	**0.24**	**0.24**	**0.20**	**0.12**	**0.18**	**0.57**	**0.94**	**0.37**	**1.57**	**1.69**	**1.64**		**0.09**	**0.44**	**3.08**
	**RSD (%)**	**13.42**	**3.67**	**15.82**	**9.79**	**20.50**	**69.23**	**24.25**	**47.04**	**7.51**	**16.70**	**24.83**	**10.96**	**25.70**	**13.78**	**20.43**	**27.08**		**3.85**	**18.06**	**18.71**
MW 1	sample 1	107.54	39.03	165.90	74.77	520.20	24.45	10.12	46.53	20.92	135.81	24.85	23.71	34.00	27.19	177.14	n.d.	n.d.	n.d.	n.d.	n.d.
	sample 2	111.69	46.71	147.16	92.30	680.35	24.28	12.00	43.56	23.47	180.85	25.25	25.88	36.84	32.89	203.15	n.d.	n.d.	n.d.	n.d.	n.d.
	sample 3	88.30	36.61	143.49	84.71	565.95	19.23	9.38	38.48	23.16	144.60	22.44	23.41	30.13	32.04	189.90	n.d.	n.d.	n.d.	n.d.	n.d.
	**Mean**	**102.51**	**40.78**	**152.18**	**83.93**	**588.83**	**22.65**	**10.50**	**42.85**	**22.51**	**153.75**	**24.18**	**24.33**	**33.65**	**30.70**	**190.06**					
	**SD**	**10.19**	**4.30**	**9.82**	**7.18**	**67.35**	**2.42**	**1.10**	**3.33**	**1.14**	**19.49**	**1.24**	**1.10**	**2.75**	**2.51**	**10.62**					
	**RSD (%)**	**9.94**	**10.55**	**6.45**	**8.55**	**11.44**	**10.69**	**10.51**	**7.76**	**5.05**	**12.68**	**5.13**	**4.52**	**8.18**	**8.18**	**5.59**					

Table S6. Statistical significance of variance between white and red grape accessions for phenolic classes

	P-Value	
Chemical class	2017	2018
HCA	0.10	0.03
HBA	0.49	0.81
Stilbenes	0.19	0.26
Flavan-3-ols	0.12	0.07
Flavonols	<0.001	<0.001
Others	0.91	0.88
Anthocyanins	<0.001	<0.001
Total polyphenols	<0.001	<0.001

Abbreviations: HBA, hydroxybenzoic acids; HCA, hydroxycinnamic acids.
P -values after analysis of variance (one-way ANOVA), α = 0.05

Table S7. Statistical significance of variance between the harvests 2017 and 2018 for phenolic classes

	P-Value	
Chemical class	red grapes	white grapes
HCA	0.87	0.25
HBA	0.26	0.01
Stilbenes	0.68	0.80
Flavan-3-ols	0.67	0.87
Flavonols	0.27	0.92
Others	0.68	0.58
Anthocyanins	0.69	0.96
Total polyphenols	0.63	0.93

Abbreviations: HBA, hydroxybenzoic acids; HCA, hydroxycinnamic acids.
P -values after analysis of variance (one-way ANOVA), α = 0.05

Table S8. Statistical significance of variance between 2017 and 2018 and between white and red grape accessions

		Statisticical significance of variance between 2017 and 2018			Statisticical significance of variance between red and white grapes	
		red	white		2017	2018
p-hydroxybenzoic acid		0.22	0.58		0.13	0.007
vanillic acid		0.76				
gallic acid		0.41	0.85		0.84	0.35
methyl gallate		0.23	0.30		0.000	0.03
ellagic acid		0.43	0.06		0.02	0.001
caftaric acid		0.25	0.01		0.44	0.73
fertaric acid		0.75	0.34		0.39	0.21
t-resveratrol		0.61	0.16		0.04	0.15
cis-resveratrol		0.53			0.55	
piceatannol		0.81	0.71		0.03	0.11
t-piceide		0.33	0.64		0.26	0.38
cis-piceide		0.84	0.90		0.43	0.44
isorhapontin		0.06	0.32		0.07	0.19
catechin		0.74	0.33		0.02	0.011
epicatechin		0.01	0.001		0.93	0.85
gallocatechin		0.43	0.05		0.20	0.03
epigallocatechin gallate		0.44	0.08		0.06	0.04
epicatechin gallate		0.94	0.56		0.024	0.006
procyanidin B1		0.20	0.20		0.009	0.03
procyanidin B2 + B4		0.11	0.31		0.53	0.14
procyanidin B3 (as B1)		0.49	0.40		0.16	0.07
quercetin			0.50		0.73	
taxifolin		0.50	0.17		0.25	0.41
myricetin		0.67	0.90		0.06	0.04
quercetin-3-rha		0.83	0.04		0.007	0.50
myricitrin		0.87				
quercetin-3-glc/gal		0.40	0.98		0.000	0.000
isorhamnetin-3-glc		0.37	0.34		0.10	0.006
syringetin-3-glc/gal		0.48	0.35		0.001	0.000
rutin		0.22	0.57		0.40	0.006
quercetin-3,4-diglc		0.31	0.60		0.08	0.346
quercetin-3-glu		0.41	0.89		0.001	0.000
kaempferol-3-glu		0.94	0.56		0.000	0.000
kaempferol-3-glc		0.11	0.67		0.001	0.000
kaempferol-3-rutinoside		0.28	0.23		0.09	0.50
isorhamnetin-3-rutinoside			0.74			
phlorizin		0.91	0.95		0.29	0.53
naringenin-7-glc		0.71	0.20		0.05	0.72
arbutin		0.41	0.74		0.30	0.38
luteolin-7-glc		0.43	0.54		0.23	0.33
Dp 3-glc		0.94			0.006	
Cn 3-glc		0.71			0.60	

Table S8 continued

Pt 3-glc		0.38			0.08	
Pn 3-glc		0.04			0.001	
Mv 3-glc		0.09				
Dp 3-acetylglc		0.80				
Cn3-acetylglc		0.78			0.53	
Pt-acetylglc		0.59				
Pn-3-acetylglc		0.78				
Mv-3-acetylglc		0.96			0.27	
Dp-3-coumclg		0.61				
Cn-3-coumclg		0.69			0.12	
Pt-3-coumclg		0.68				
Pn-3-coumclg		0.23				
Mv-3-coumclg		0.79				
MV 3,5-diglc		0.30				
DP 3,5-diglc		0.82				
CN 3,5-diglc		0.95				
PT 3,5-diglc		0.95				
PN 3,5-diglc		0.64				

P-values after analysis of variance (one-way ANOVA), $\alpha = 0.05$

Table 9a. Concentrations of phenolic classes found in grapevine hybrids of different breeding origins harvested in 2017 (in mg kg^{-1} FW)

Number of accessions	Origin of accessions	Grape colour	HBA[a]	HCA[a]	Stilbenes[a]	Flavan-3-ols[a]	Flavonols[a]	Others[a]	Anthocyanins[a]	Total[a]
1	Austria	blanc	**27.7**	**76.9**	**1.1**	**779.9**	**95.3**	**4.7**	**0.0**	**985.6**
10	Breeding line (InnoVitis, Italy)	blanc	**26.1** ± 15.7	**40.7** ± 21.5	**14.9** ± 22.6	**673.4** ± 272.7	**139.9** ± 56.4	**3.3** ± 0.9	**1.9** ± 5.7	**900.2** ± 323.5
1	France	blanc	**20.5**	**10.4**	**3.6**	**134.2**	**143.8**	**1.4**	**0.0**	**313.8**
2	Germany	blanc	**11.8** ± 1.9	**19.2** ± 5.1	**1.6** ± 0.6	**550.0** ± 346.4	**88.1** ± 0.7	**3.0** ± 0.9	**22.1** ± 22.1	**695.7** ± 333.6
6	Hungary	blanc	**12.7** ± 2.5	**21.9** ± 10.8	**4.0** ± 3.5	**501.3** ± 196.4	**67.8** ± 19.8	**2.1** ± 1.0	**0.0** ± 0.0	**609.8** ± 220.4
12	Progeny individuals (InnoVitis, Italy)	blanc	**35.8** ± 12.3	**46.8** ± 24.3	**3.5** ± 2.4	**934.3** ± 344.5	**168.3** ± 38.0	**3.6** ± 0.8	**17.0** ± 25.1	**1209.3** ± 354.9
3	Serbia	blanc	**26.3** ± 15.0	**35.7** ± 8.3	**3.9** ± 3.6	**436.1** ± 135.1	**135.5** ± 25.4	**2.5** ± 0.7	**0.0** ± 0.0	**640.0** ± 165.6
1	Austria	noir	**22.5**	**22.6**	**37.0**	**577.1**	**93.2**	**3.3**	**1432.1**	**2187.8**
7	Breeding line (InnoVitis, Italy)	noir	**22.8** ± 11.4	**32.1** ± 17.7	**13.9** ± 9.7	**470.0** ± 247.8	**75.8** ± 30.8	**2.1** ± 0.7	**1473.1** ± 586.5	**2089.8** ± 639.0
5	Czech republic	noir	**14.8** ± 4.5	**33.1** ± 22.2	**7.2** ± 4.4	**410.7** ± 131.5	**73.8** ± 45.8	**3.9** ± 1.0	**2692.6** ± 1725.3	**3236.2** ± 1829.6
2	France	noir	**16.7** ± 2.7	**55.2** ± 29.4	**10.1** ± 3.3	**630.2** ± 28.1	**51.6** ± 26.3	**3.6** ± 0.7	**1463.7** ± 35.0	**2231.0** ± 125.5
1	Germany	noir	**27.4**	**101.3**	**4.9**	**1159.2**	**87.2**	**3.1**	**963.6**	**2346.8**
1	Hungary	noir	**16.9**	**10.3**	**14.4**	**278.2**	**47.5**	**1.6**	**1378.6**	**1747.5**
5	Progeny individuals (InnoVitis, Italy)	noir	**23.6** ± 9.9	**51.7** ± 32.2	**8.2** ± 10.8	**762.3** ± 237.0	**127.7** ± 31.3	**3.8** ± 0.9	**1459.1** ± 489.7	**2436.5** ± 383.8
1	Russia	noir	**16.9**	**96.7**	**3.8**	**417.7**	**37.0**	**2.1**	**1810.9**	**2385.1**

[a]Mean ± standard deviation.

Abbreviations: HBA, hydroxybenzoic acids; HCA, hydroxycinnamic acids.

Table 9b. Concentrations of phenolic classes found in grapevine hybrids of different breeding origins harvested in **2018** (in mg kg^{-1} FW)

Number of accessions	Origin of accessions	Grape colour	HBA[a]	HCA[a]	Stilbenes[a]	Flavan-3-ols[a]	Flavonols[a]	Others[a]	Anthocyanins[a]	Total[a]
1	Austria	blanc	**21.6**	**123.9**	**3.7**	**711.6**	**86.7**	**3.8**	**0.0**	**951.2**
10	Breeding line (InnoVitis, Italy)	blanc	**35.2** ± 16.0	**57.8** ± 20.9	**17.0** ± 29.2	**848.0** ± 293.5	**162.8** ± 78.3	**3.6** ± 1.1	**3.1** ± 9.2	**1127.4** ± 364.5
1	France	blanc	**21.9**	**19.0**	**9.0**	**198.7**	**115.4**	**2.0**	**0.0**	**365.9**
2	Germany	blanc	**17.8** ± 5.8	**50.9** ± 9.7	**0.5** ± 0.2	**334.5** ± 249.5	**136.1** ± 7.6	**3.4** ± 0.8	**23.5** ± 23.5	**566.6** ± 249.8
6	Hungary	blanc	**15.0** ± 6.0	**36.8** ± 23.6	**4.6** ± 2.5	**438.5** ± 371.6	**82.2** ± 42.1	**1.8** ± 1.1	**0.0** ± 0.0	**578.9** ± 403.3
12	Progeny individuals (InnoVitis, Italy)	blanc	**38.3** ± 11.3	**50.4** ± 21.0	**4.2** ± 3.4	**837.4** ± 183.9	**141.0** ± 61.0	**3.9** ± 1.0	**15.1** ± 25.0	**1090.2** ± 201.1
3	Serbia	blanc	**28.2** ± 15.4	**75.3** ± 21.0	**2.3** ± 1.7	**348.3** ± 33.5	**136.6** ± 47.5	**2.6** ± 0.5	**0.0** ± 0.0	**593.2** ± 108.7
1	Austria	noir	**29.5**	**32.1**	**28.3**	**690.6**	**149.2**	**3.9**	**1326.5**	**2260.0**
7	Breeding line (InnoVitis, Italy)	noir	**23.4** ± 18.1	**45.8** ± 18.2	**11.0** ± 9.8	**525.3** ± 347.5	**71.8** ± 33.5	**2.6** ± 0.6	**1425.4** ± 634.4	**2105.2** ± 548.7
5	Czech republic	noir	**7.7** ± 1.2	**55.8** ± 68.1	**12.8** ± 17.6	**390.4** ± 124.5	**62.6** ± 25.2	**4.0** ± 1.4	**2476.7** ± 2007.4	**3010.0** ± 2188.0
2	France	noir	**25.8** ± 7.9	**67.8** ± 6.7	**28.8** ± 10.1	**601.3** ± 47.2	**80.8** ± 49.8	**2.9** ± 0.5	**2239.1** ± 107.7	**3046.4** ± 15.8
1	Germany	noir	**11.9**	**118.7**	**2.1**	**778.7**	**70.2**	**4.4**	**772.6**	**1758.6**
1	Hungary	noir	**6.2**	**29.1**	**6.5**	**123.5**	**35.9**	**1.1**	**1212.9**	**1415.2**
5	Progeny individuals (InnoVitis, Italy)	noir	**29.0** ± 34.1	**53.3** ± 34.1	**10.2** ± 8.0	**640.4** ± 207.7	**67.2** ± 24.0	**3.4** ± 0.9	**995.8** ± 306.1	**1799.4** ± 281.7
1	Russia	noir	**3.9**	**99.4**	**1.3**	**438.7**	**43.8**	**3.7**	**1397.8**	**1988.6**

[a]Mean ± standard deviation.

Abbreviations: HBA, hydroxybenzoic acids; HCA, hydroxycinnamic acids.

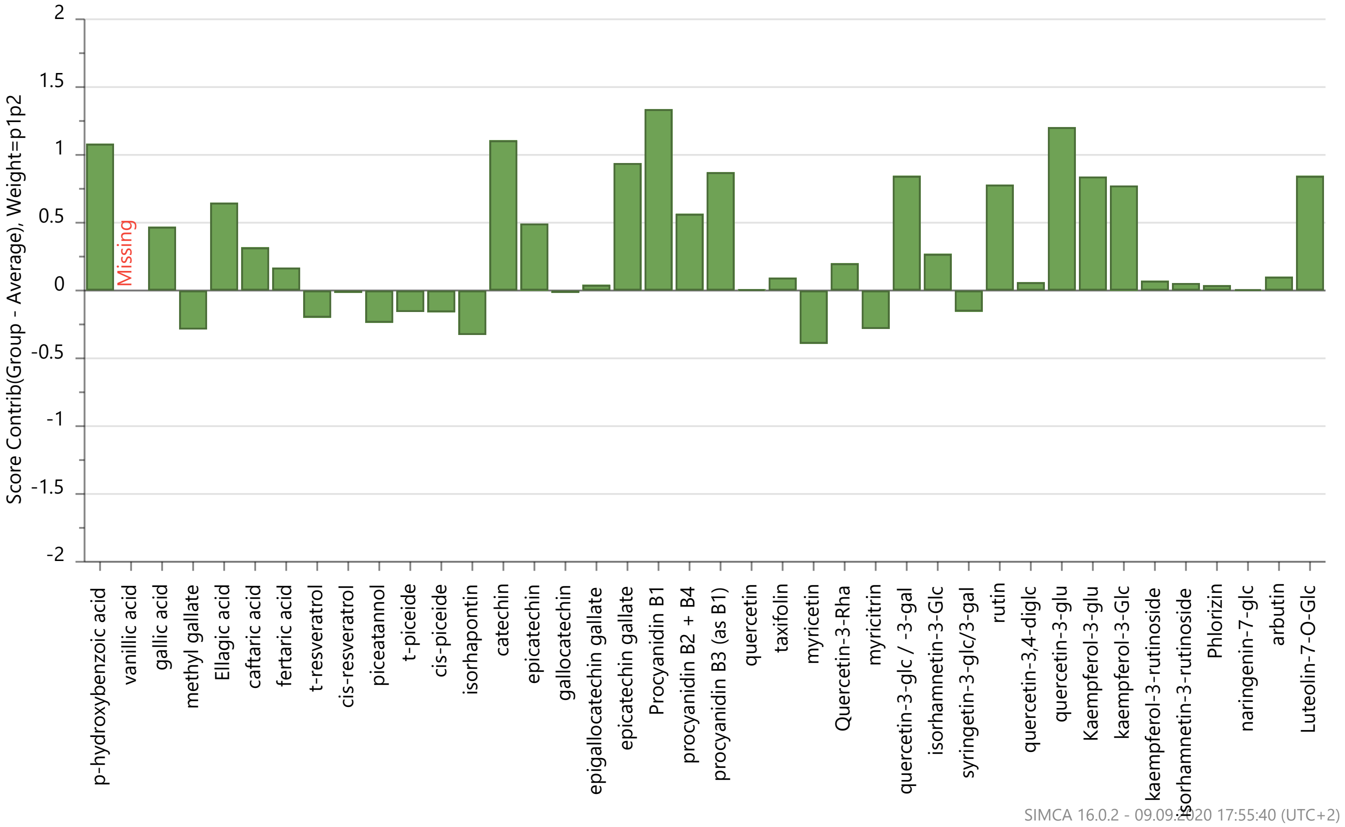

Figure S1. PCA contribution plot of white grapes versus average (anthocyanins excluded)

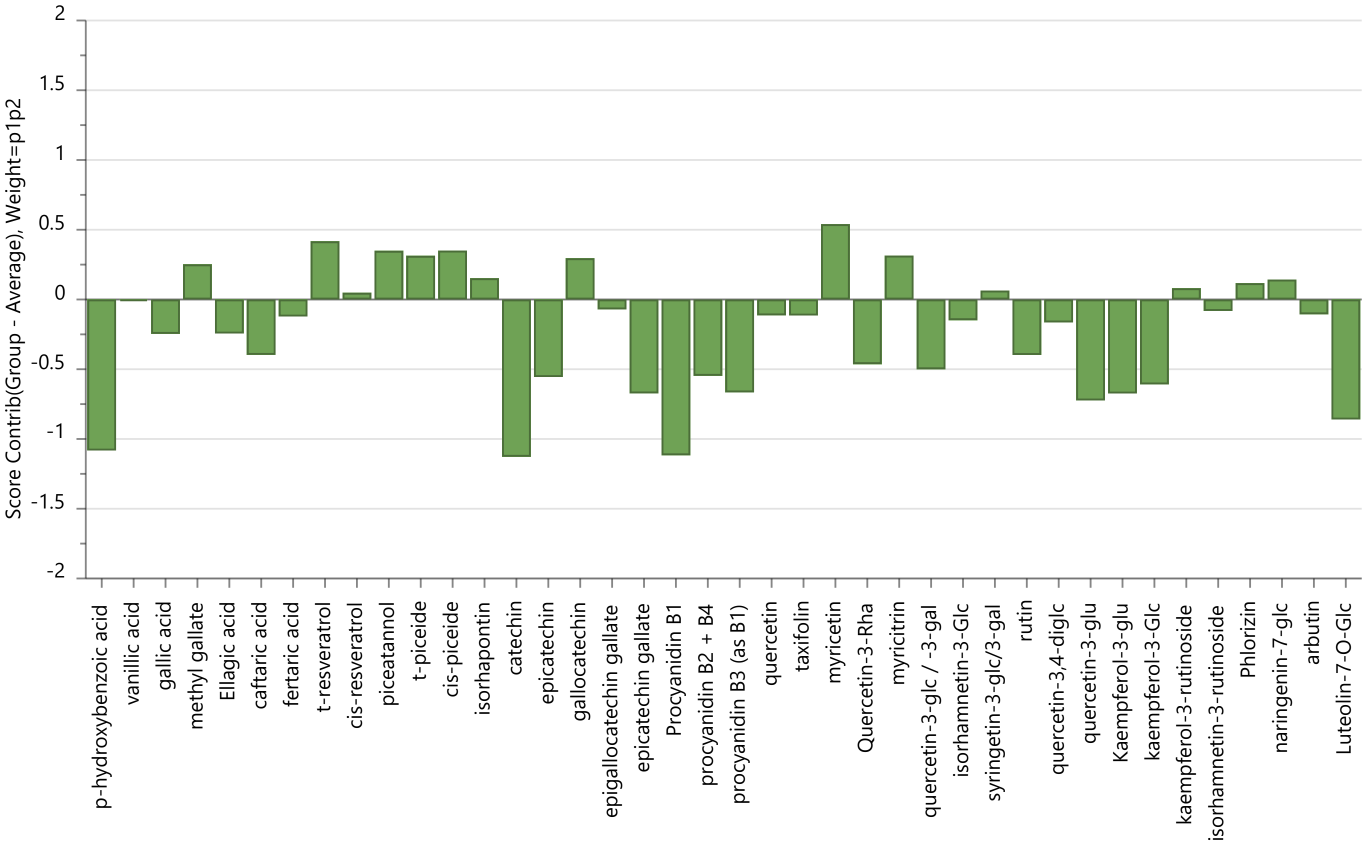

Figure S2. PCA contribution plot of red grapes versus average (anthocyanins excluded)

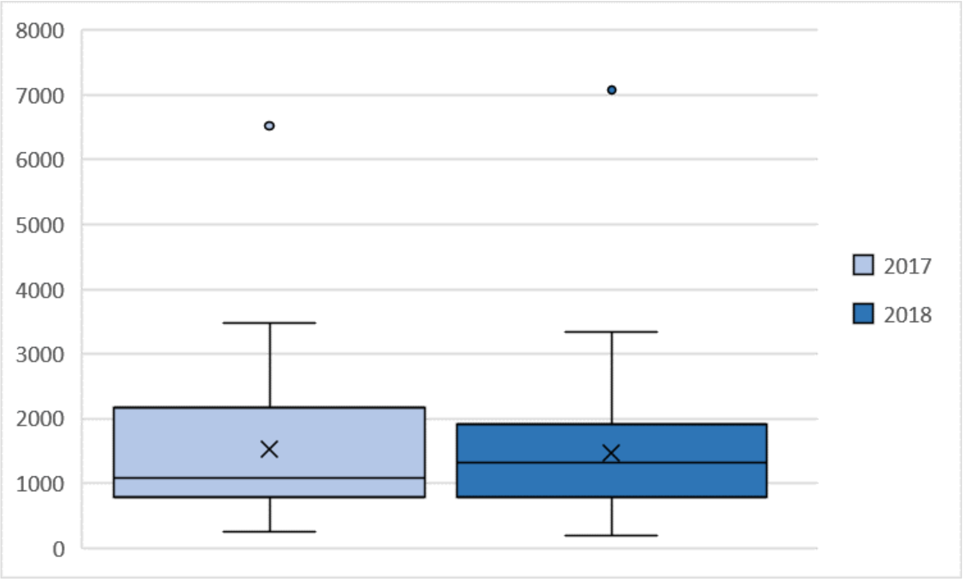

Figure S3. Box plot illustrating the total phenolic content in all accessions (red and white grapes) in 2017 and 2018 (Description of boxes: Median = central line, Mean = cross, interquartile range box = 25th to 75th percentile, whiskers = less than 1.5 times interquartile range, outliers marked as filled circles).

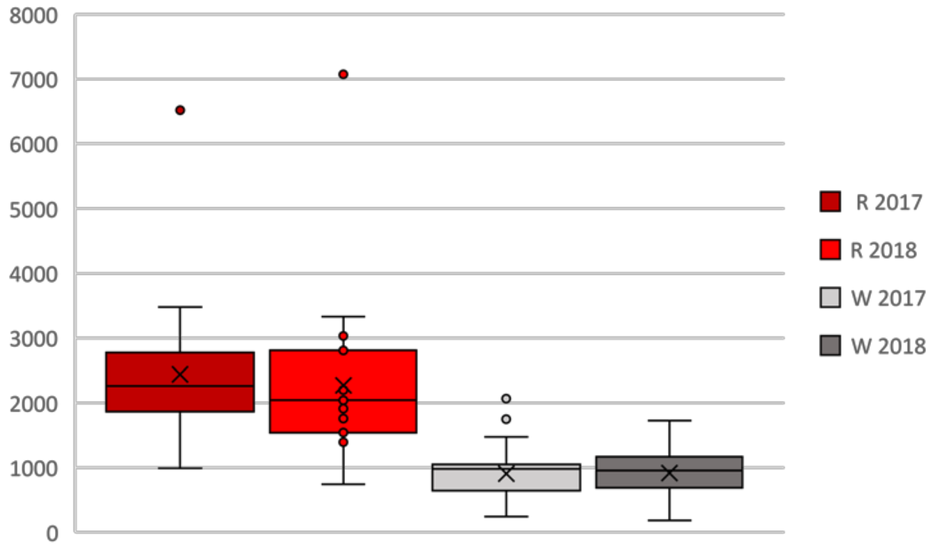

Figure S4. Box plot illustrating the total phenolic content in red (R) and white (W) grapes in 2017 and 2018 (Description of boxes: see figure S3).

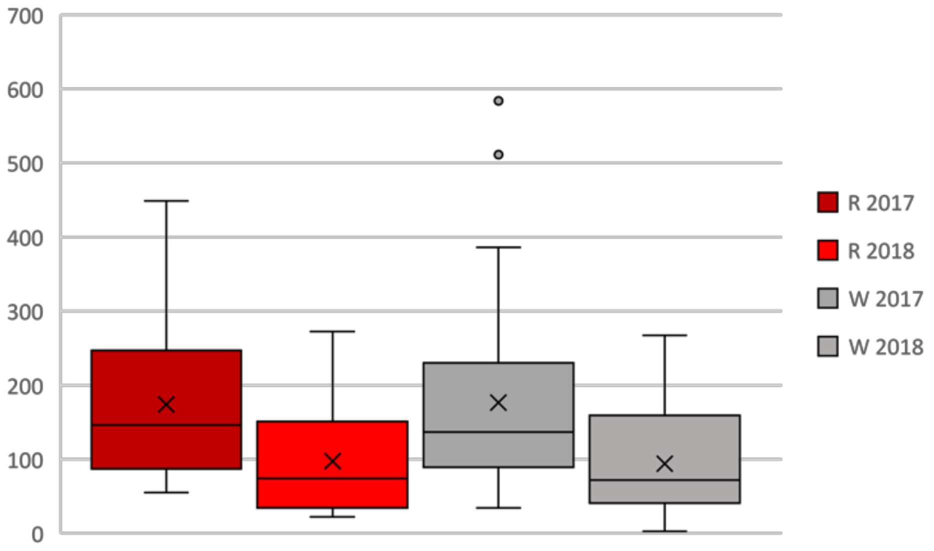

Figure S5. Box plot illustrating the content of epicatechin n red (R) and white (W) grapes in 2017 and 2018 (Description of boxes: see figure S3).

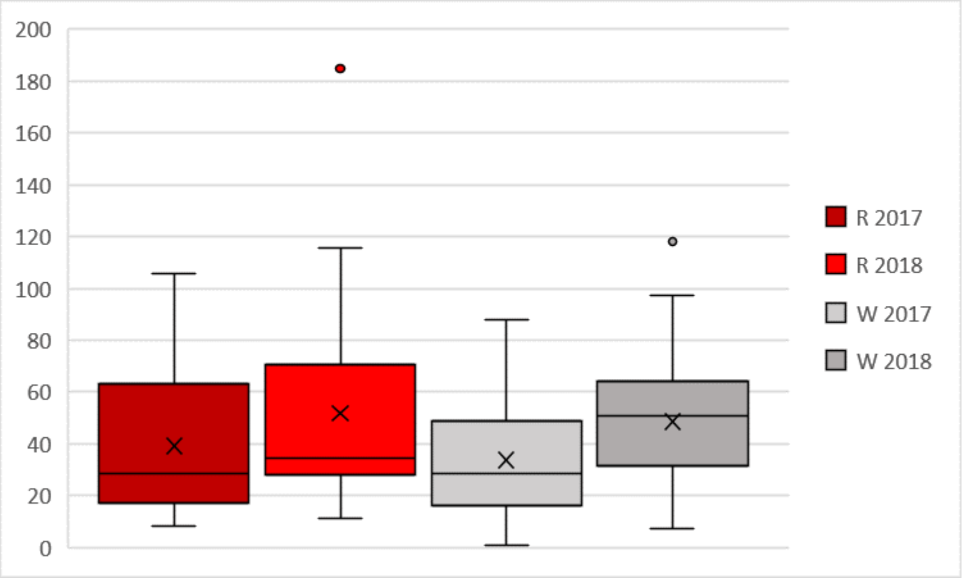

Figure S6. Box plot illustrating the content of caftaric acid n red (R) and white (W) grapes in 2017 and 2018 (Description of boxes: see figure S3).

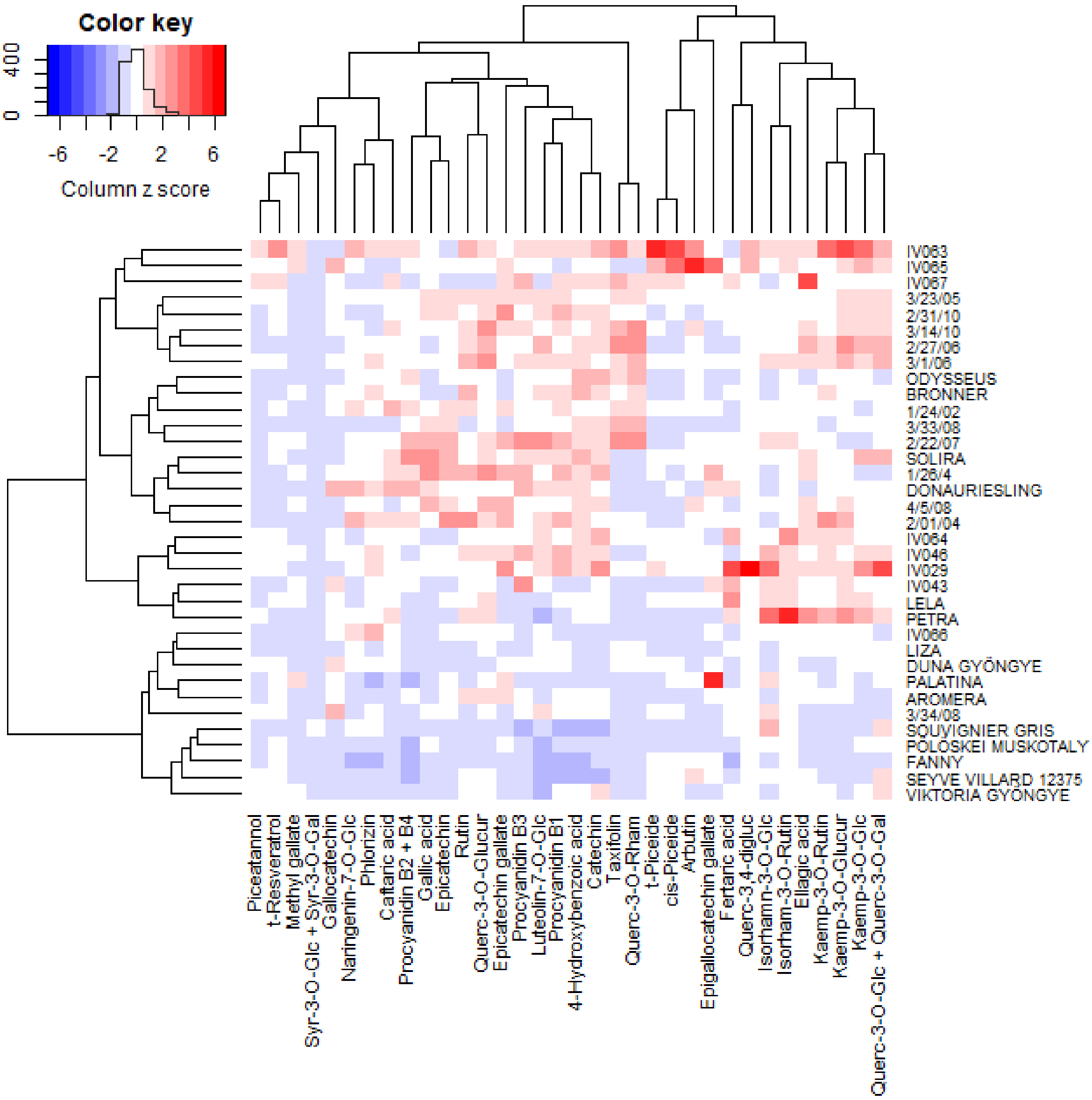

Figure S7. Heatmap of white grapevine accessions and identified compounds in two consecutive years (2017 and 2018). Data were mean-centered, and unit-variance scaled prior to the calculation of the dendrograms. Dendrograms were constructed by hierarchical clustering analysis using "Canberra" as the distance function and Ward.D2 as the hierarchical clustering function.

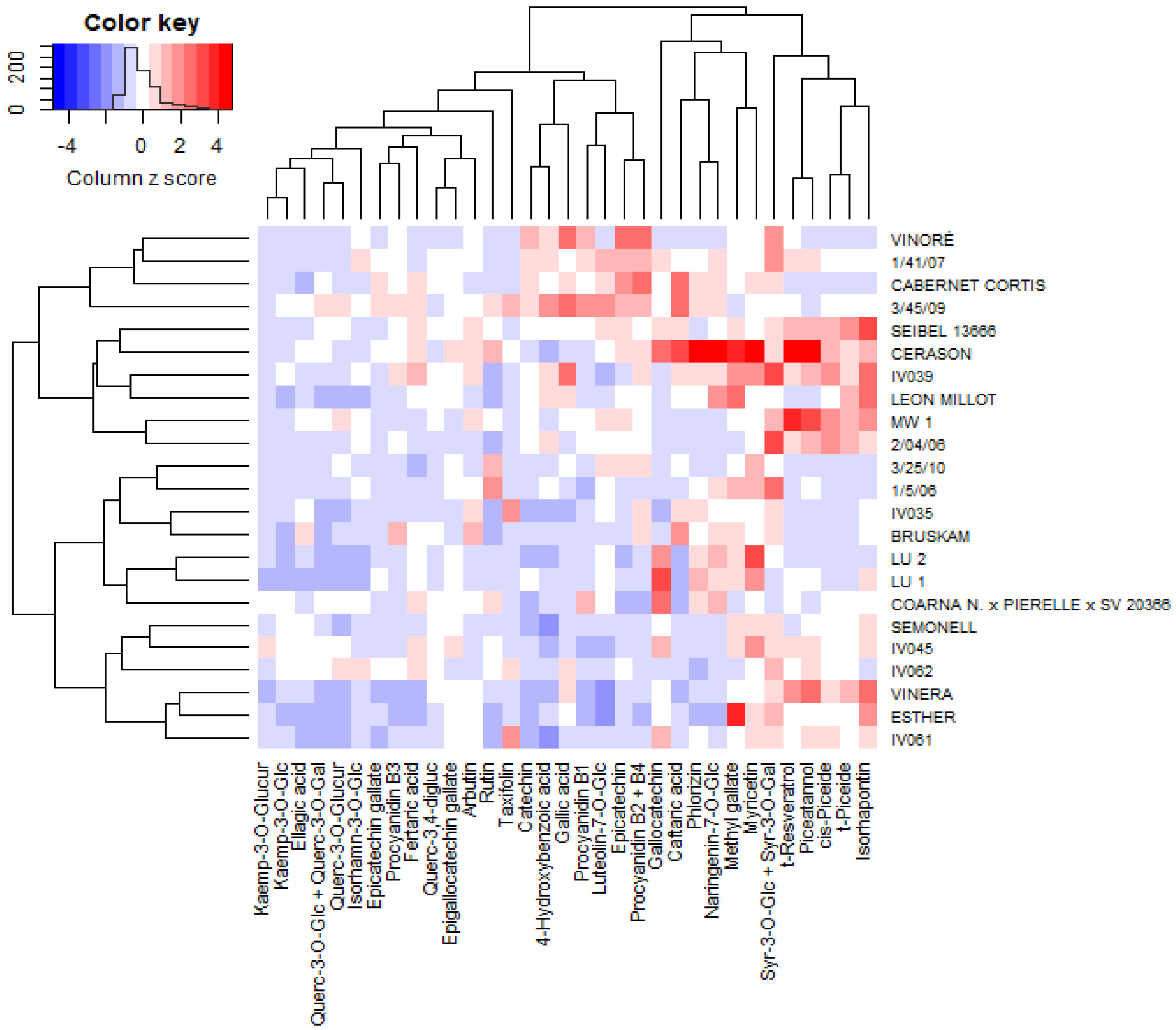

Figure S8. Heatmap of red grapevine accessions and identified compounds in two consecutive years (2017 and 2018). Data were mean-centered, and unit-variance scaled prior to the calculation of the dendrograms. Dendrograms were constructed by hierarchical clustering analysis using “Canberra” as the distance function and Ward.D2 as the hierarchical clustering function.
